# Single-cell transcriptomic analysis of zebrafish cranial neural crest reveals spatiotemporal regulation of lineage decisions during development

**DOI:** 10.1016/j.celrep.2021.110140

**Published:** 2021-12-21

**Authors:** David Tatarakis, Zixuan Cang, Xiaojun Wu, Praveer P. Sharma, Matthew Karikomi, Adam L. MacLean, Qing Nie, Thomas F. Schilling

**Affiliations:** 1 Department of Developmental and Cell Biology, University of California, Irvine, Irvine, CA, USA; 2 Department of Mathematics, University of California, Irvine, Irvine, CA, USA; 3 Department of Quantitative and Computational Biology, University of Southern California, Los Angeles, CA, USA; 4 Lead contact

## Abstract

Neural crest (NC) cells migrate throughout vertebrate embryos to give rise to a huge variety of cell types, but when and where lineages emerge and their regulation remain unclear. We have performed single-cell RNA sequencing (RNA-seq) of cranial NC cells from the first pharyngeal arch in zebrafish over several stages during migration. Computational analysis combining pseudotime and real-time data reveals that these NC cells first adopt a transitional state, becoming specified mid-migration, with the first lineage decisions being skeletal and pigment, followed by neural and glial progenitors. In addition, by computationally integrating these data with RNA-seq data from a transgenic Wnt reporter line, we identify gene cohorts with similar temporal responses to Wnts during migration and show that one, Atp6ap2, is required for melanocyte differentiation. Together, our results show that cranial NC cell lineages arise progressively and uncover a series of spatially restricted cell interactions likely to regulate such cell-fate decisions.

## In brief

Tatarakis et al. provide a single-cell transcriptomic timeline of cranial neural crest (NC) development in zebrafish and address long-standing questions surrounding the integration of NC cell migration and lineage specification. They find that lineages are specified mid-migration. These fate decisions correspond to shifts in Wnt signaling, and lineages rapidly segregate.

## Graphical Abstract

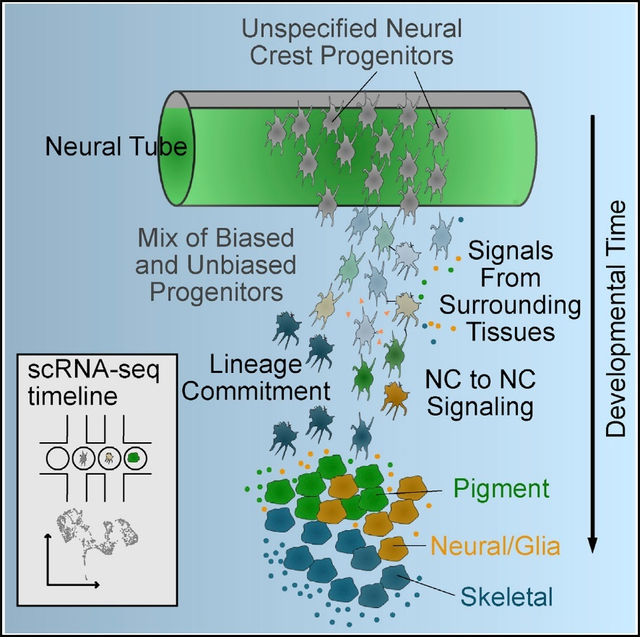

## INTRODUCTION

A fundamental question in developmental biology is how embryonic cells acquire their fates as part of tissues undergoing rapid growth and cell rearrangements. A dramatic example of this in vertebrates is the neural crest (NC), a highly migratory and multipotent transient cell population. NC cells are specified at the border of the neuroepithelium and epidermis, undergo an epithelial-mesenchymal transition (EMT), and migrate extensively throughout the embryo to generate a wide variety of cell types, including cartilage, bone, neurons, glia, pigment, and many others (Le Douarin, 1980; Weston, 1970). Despite many studies addressing the origins and emergence of these cell types during NC migration, the degree to which individual NC cells are multipotent or biased toward particular lineages, as well as the timing of their lineage decisions, remains unclear (Dupin et al., 2018; Kalcheim and Kumar, 2017; Prasad et al., 2019).

Lineage-tracing experiments *in vivo* have produced evidence both for NC multipotency as well as early lineage restrictions prior to migration. Cell-labeling experiments in the chick have shown that single premigratory NC cells can give rise to multiple fates (Bronner-Fraser and Fraser, 1988; Bronner-Fraser et al., 1980; McKinney et al., 2013), while in zebrafish, many single labeled cranial NC cells generate cell-type-restricted clones, depending on their initial premigratory locations (Schilling and Kimmel, 1994). Cell labeling of trunk NC cells in chick and zebrafish embryos has similarly provided evidence for a spatiotemporal map of NC cell fate independent of migratory environment (Krispin et al., 2010; Raible and Eisen, 1994), although lineage tracing in mice using genetically encoded fluorescent tags suggests that early NC cells show no apparent lineage restrictions (Baggiolini et al., 2015). A variety of *in vitro* studies, for example, serially isolating single NC cells and assaying the array of cell types they can generate in culture, have also shown restricted potency (Stemple and Anderson, 1992). These seemingly inconsistent findings highlight the need for further investigation into the timing and mechanisms of lineage specification in the NC.

Recent studies have revisited this issue with single-cell transcriptomic and epigenomic approaches. Single-cell RNA sequencing (scRNA-seq) and ATAC-seq data from fluorescence-activated cell sorting (FACS) of premigratory and migrating NC cells in mice suggest that glial and ectomesenchymal lineages arise during migration (Soldatov et al., 2019), although similar studies of chick NC cells suggest an earlier split between neural and non-neural lineages (Williams et al., 2019). Interestingly, some of the single-cell evidence points to a population of stem-like premigratory NC cells in zebrafish (Lukoseviciute et al., 2018), and this is supported by spatial genomic analyses of gene expression in the chick dorsal neural tube (NT) (Lignell et al., 2017). However, another independent single-cell NC study in chick shows no evidence for similar early stem-like subpopulations (Morrison et al., 2017). One major limitation of all of these studies is the lack of temporal information. Global changes in gene expression as NC cells delaminate and migrate are difficult to deconvolve from expression signatures that indicate lineage bifurcation when looking at a single snapshot in time. Further, lineage decisions likely span a range of developmental time, making it unlikely that all will be captured at one time point. Finally, computational methods for trajectory inference (pseudotime and RNA velocity), while powerful, are best used as hypothesis-generating tools. When used in a vacuum, they can sometimes imply lineage connections and differentiation that does not comport with experimentation. Thus, many questions remain as to precisely when and where distinct cell lineages emerge in the NC, as well as the mechanisms that regulate their fate decisions.

To address these questions, we have generated scRNA-seq datasets for cranial NC cells that contribute to the first pharyngeal arch (PA1) in zebrafish. Unlike previous single-cell NC studies, which lack spatial information and rely on pseudotime to infer temporal changes in gene expression, our study focuses on the PA1 subpopulation over a closely spaced series of stages (every few hours) and computational integration of pseudotime and real-time data. Our scRNA-seq data suggest that premigratory NC cells destined for PA1 are relatively homogeneous at the transcriptional level. Gene expression signatures of pigment and skeletal lineages emerge mid-migration, and a neural and glial signature becomes apparent slightly later during migration. We identify several markers of these lineages and confirm their expression and spatial segregation within the migrating NC using *in situ* hybridization. Emergence of these lineage-specific gene-expression signatures correlates with shifts in Wnt signaling between NC cells and surrounding tissues, which we show by computationally integrating bulk RNA-seq data from Wnt reporters with the scRNA-seq data. Our findings provide valuable insights into the specification of lineages in the cranial NC and uncover regulators of cell fate decisions.

## RESULTS

### scRNA-seq of cranial NC cells reveals heterogeneity in gene expression during migration

**Figure 1.**
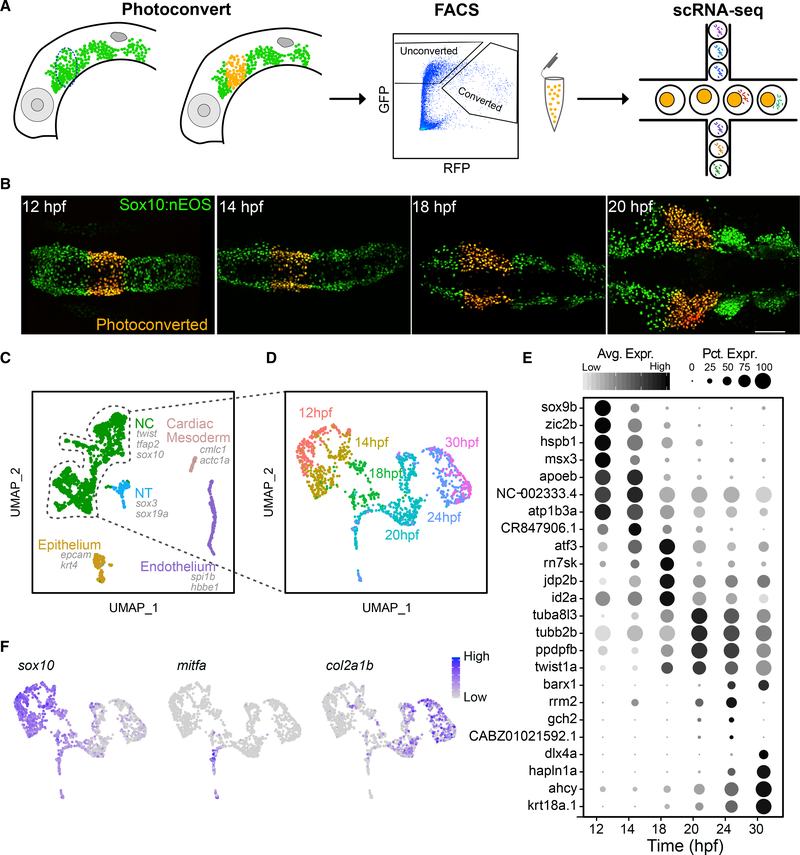
Time course scRNA-seq of isolated first pharyngeal arch (PA1) neural crest (NC) cells (A) Diagram showing experimental design. Lateral views of the head of a *sox10:nEOS* transgenic, showing migrating NC cells expressing photoconvertible nEOS (green) that were photoconverted (red) and isolated via FACS and then sequenced using 10X Genomics Drop-seq. (B) Live fluorescent images, dorsal views, anterior to the left, showing photoconverted NC cells in the PA1 migratory stream at 4 of 6 time points. (C) Unbiased clustering and UMAP embedding showing NC and other cell types isolated. All 6 time points were aggregated and analyzed together. Canonical markers for each cell type are displayed. (D) The NC subpopulation from (C) was subclustered and reanalyzed. The UMAP displays time point identities for each cell. (E) Dot plot displaying markers for each time point and their relative levels of expression. Size of dots represents percentage of cells expressing the gene. Gray scale intensity represents relative average expression among cells. (F) UMAPs displaying relative expression levels for presumptive early NC, pigment, and cartilage lineages marked by *sox10*, *mitfa*, and *col2a1b,* respectively. For micrographs, scale bar represents 100 mm. Each time point represents 1 library made up of 6–8 embryos.

To profile heterogeneity in gene expression over time in a defined population of cranial NC cells, we constructed a single-cell transcriptomic timeline for cells that contribute to the PA1 over the period that they migrate. We isolated cells of the PA1 migratory stream using a transgenic photoconvertible marker of NC cells, *tg(sox10:nEOS)* (Figure 1A). Photoconverted cells were isolated via FACS at six embryonic stages from the onset to essentially completion of NC migration in this stream: 12; 14; 18; 20; 24; and 30 h post-fertilization (hpf) (Figure 1B). Single-cell cDNA libraries were then constructed via the 10X Genomics platform and sequenced. Low-quality cells were excluded based on numbers of distinct genes detected and percentages of reads mapped to mitochondrial genes (Figures S1A–S1D). Clustering and marker gene analysis using Seurat v3 (Stuart et al., 2019) revealed a large NC population and a few smaller populations of likely non-NC cell types, including endodermal, mesodermal and cardiac, neuroectodermal, and epithelial progenitors (Figures 1C and S1E–S1G). These presumptive non-NC cells were removed from further analyses to obtain a pure population of NC cells. Following clustering and dimensionality reduction using Seurat, these cells largely clustered within their time point identities (Figure 1D). Marker genes obtained through differential gene expression analysis using Seurat revealed general trends in temporal gene expression dynamics (Figure 1E). For example, expression of early NC specification genes, including *sox10*, was very strong in the 12 and 14 hpf populations but considerably reduced later. Conversely, expression of many pigment specifiers, including *microphthalmia transcription factor* (*mitfa*), as well as markers of skeletal lineages, including *collagen type2a1b* (*col2a1b*), progressively increased at mid-late migration stages (Figure 1F).

**Figure 2.**
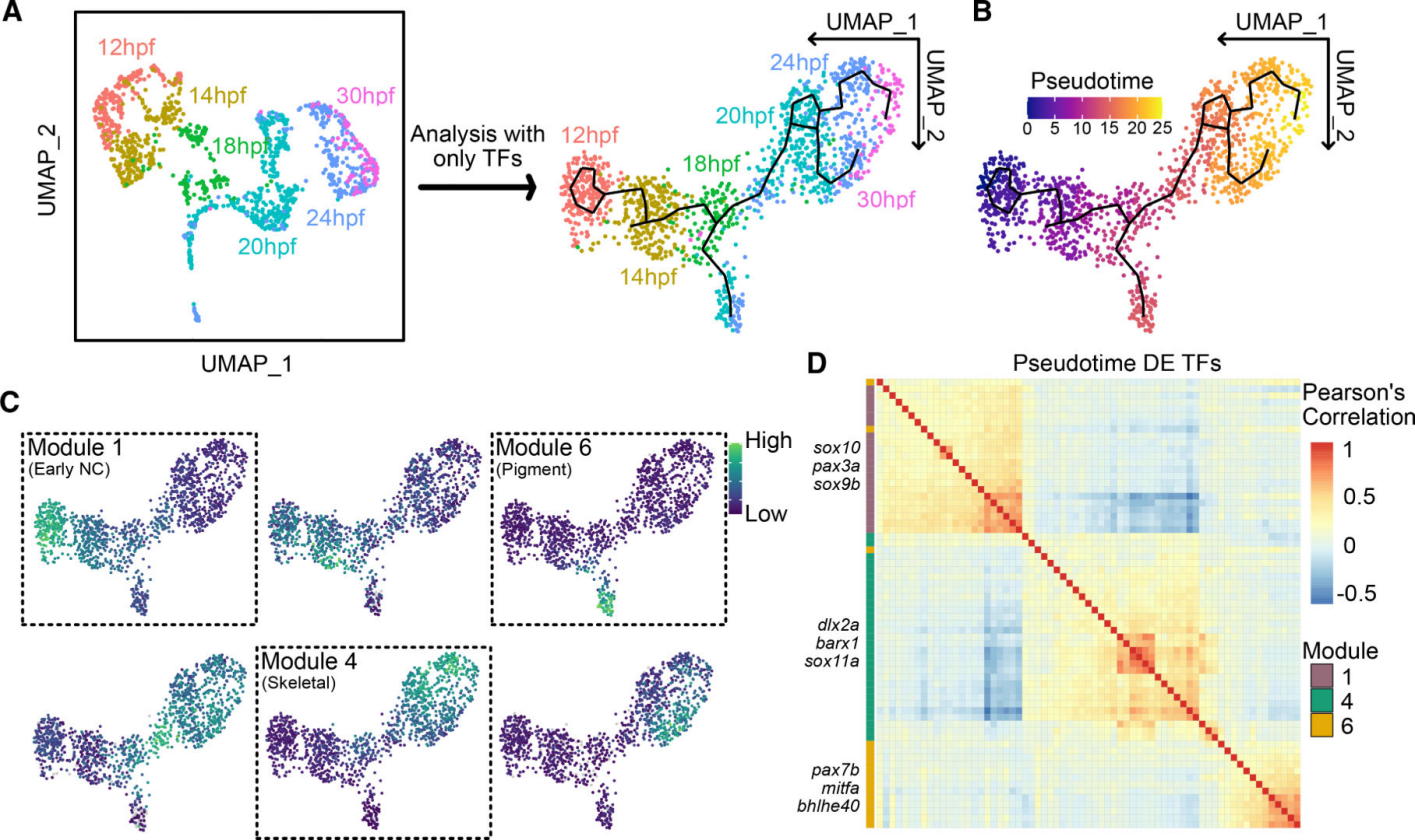
Temporal analysis of transcription factors in the NC (A) NC dataset from Figure 1D re-analyzed using only TFs. The new UMAP shows the same broad structure but smoothens connections between time points (black lines). (B) The same UMAP overlayed with pseudotime values calculated using Monocle3. (C) Six modules of TFs identified with distinct expression profiles across pseudotime. Feature plots showing expression scores for each module overlayed with the UMAP. Three modules correspond to early NC, skeletal, and pigment branches, labeled as modules 1, 4, and 6, respectively (dashed line boxes), totaling 91 TFs. (D) Heatmap showing correlation between TFs making up early NC, skeletal, and pigment modules. Hierarchical clustering reveals groups of co-expressed TFs. Selected representative genes are labeled along the side.

Next, we aimed to investigate the broad transcriptional changes occurring across development of the PA1 NC migratory stream in more detail. Differentiation of lineages is characterized by their expression of transcription factors (TFs) that in turn drive expression of downstream differentiation programs. For example, in the pigment lineage of NC, master regulatory TFs, like Mitf, precede expression of later enzymes involved in pigment production, such as tyrosinase. Therefore, a temporal analysis of TFs can inform the specification of lineages prior to their terminal differentiation. In order to investigate the mechanisms driving the emergence of the pigment and skeletal developmental branches, we used pseudotime analysis driven by differentially expressed TFs. Dimensionality reduction and clustering with a comprehensive list of TFs (adapted from Vaquerizas et al., 2009; Figure 2A) resulted in smooth connections between clusters, which enabled us to perform trajectory inference. Combined lineage trajectory and pseudotime analysis using Monocle3 (Cao et al., 2019; Trapnell et al., 2014) identified TFs with differential expression across developmental time (Figure 2B). Louvain clustering uncovered six distinct modules of gene expression, in total consisting of 91 TFs (Figure 2C; Table S1). Three of these TF modules distinctly overlapped with the early NC, pigment, and skeletal populations (Figures 2C and 2D). Using these TFs along with other, established canonical markers, we built lists of genes that mark early NC, pigment, skeletal, and neural and glial lineages (Table S2).

### Distinct gene expression signatures arise progressively during NC migration

**Figure 3.**
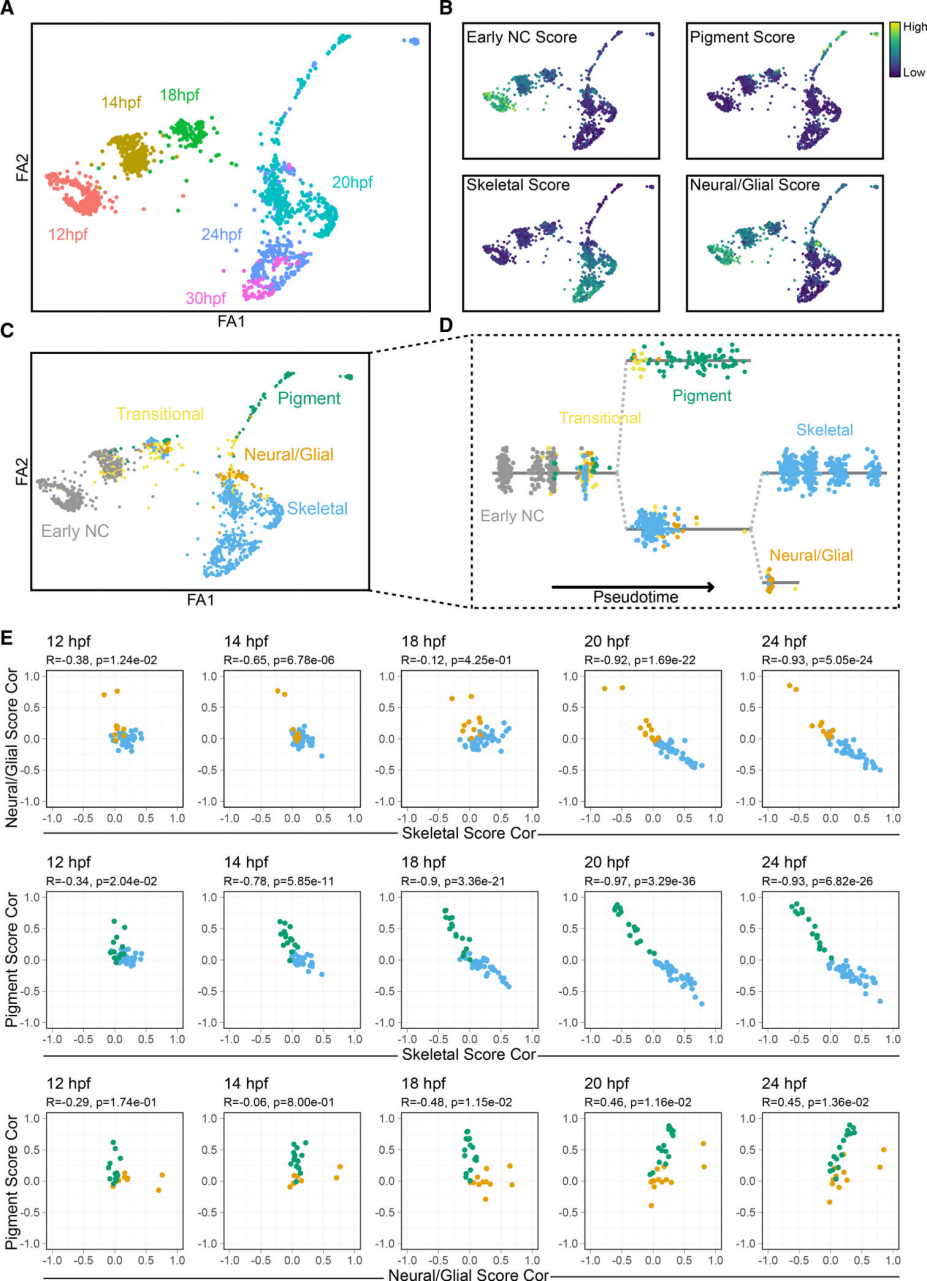
Pseudotemporal analysis reveals emergence of NC lineages during migration (A) ForceAtlas (FA) embedding of single-cell timeline initialized using PAGA graph with time point identities of individual cells labeled and color coded. (B) Module scores, color code distinct from (A), for early NC, pigment, skeletal, and neural and glial lineages overlaid on FA embedding. (C) CellAssign probabilistic cell type labels computed using module score genes overlaid on FA embedding. Color code is distinct from (A) or (B). (D) Pseudotime calculated using PAGA graph. Cells are arranged according to pseudotime values along the x axis, with pseudotime scaled within each branch. Branch points are split on the y axis and connected by dashed lines. Color code corresponds to (C). (E) Scatterplots showing the increased specificity of marker genes to lineages across developmental time. Each point represents a gene contributing to a lineage score (color code from C) with the axes showing its Pearson correlation to the total score for pigment, skeletal, or neural and glial across all cells in each time point.

To examine the temporal emergence of lineages in more detail, we utilized the partition-based graph abstraction (PAGA) method, in this case performed on the entire expression matrix. PAGA determines topology of the data and computes a measure of cell connectivity (Wolf et al., 2019). The PAGA graph was then used to initialize force-directed layout in ForceAtlas2 (Jacomy et al., 2014) for single-cell embedding that preserves global topology and connectivity (Figure 3A). Module scoring driven by our lineage marker lists (Table S2) revealed groups of cells enriched for markers of each lineage (Figure 3B). Module scores for early NC and neural and glial lineages showed similar enrichment patterns, reflective of the overlapping roles for certain factors in early NC development and later neural and glial differentiation (e.g., *foxd3* and *sox10*), and pigment progenitors also showed a medium neural and glial score. Therefore, to disentangle lineage identities, we applied probabilistic cell identification using CellAssign driven by all these lineage markers (Zhang et al., 2019). This analysis revealed cells identified as early NC, pigment, skeletal, neural and glial, and another group that belonged to none of these lineages, which we labeled as “transitional” (Figure 3C). Expression of most markers was restricted to single lineages, though a few were shared between lineages (Figure S2A). Transitional cells showed a distinct expression profile, distinct both from any specific lineage as well as earlier NC cells. PAGA embedding and connectivity were then used to calculate pseudotime and identify branch points. Importantly, when ordered by pseudotime, the cells analyzed in this manner arranged in a way that largely recapitulated real developmental time (Figure S2B), suggesting that branch points correspond closely to the temporal emergence of each lineage (Figure 3D). The temporal trajectories of NC cells inferred from these scRNA-seq data were very robust to different parameter combinations, including numbers of principal components, neighbors, and variable genes (Figures S3A–S3C). These consistently identified branching of skeletal and pigment progenitors at 18 hpf. Because markers of neural and glial progenitors were largely restricted to the 24-hpf dataset, although many markers of neighboring skeletal progenitors were expressed at 20 hpf, we conclude that the neural and glial progenitor is a terminal cell type in the inferred trajectory. In addition, although integration of each library to reduce sample-specific effects removed much of the time point expression differences, separation of lineages as well as the pigment-skeletal branching was preserved (Figure S3D). These analyses suggest that subpopulations with gene expression signatures of pigment and skeletal lineages appear first, while the neural and glial progenitor subpopulation arises later.

The dynamic specificity of lineage expression modules can be observed by examining the inter- and intra-module correlations for individual genes within a module across developmental time. We observed that, between skeletal and either pigment or neural and glial branches, intra-module correlations became more coordinated while inter-module correlations became repulsed across developmental time points. This trend was less pronounced between the pigment and neural and glial branches (Figure 3E).

**Figure 4.**
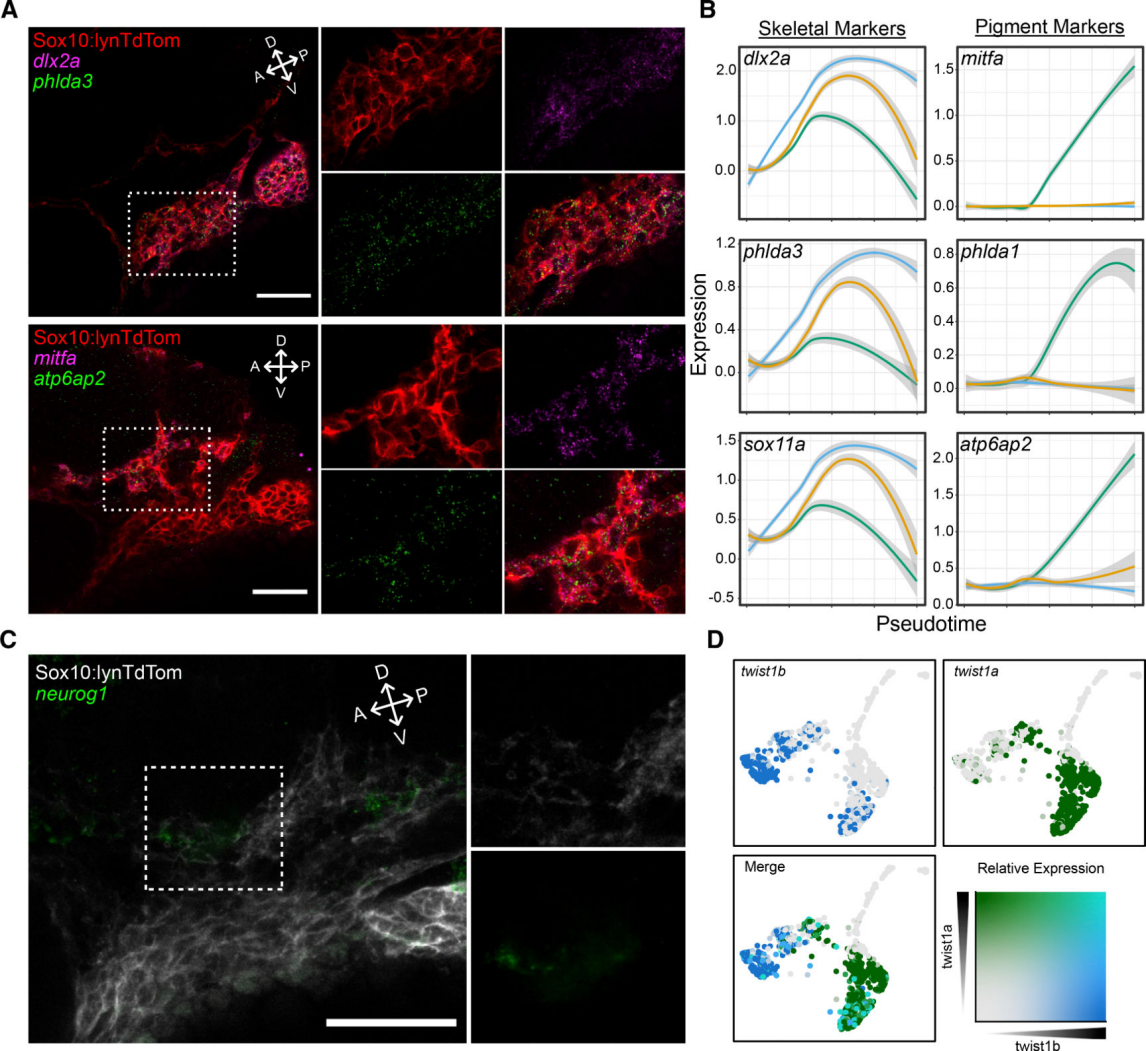
*In situ* hybridization chain reaction (isHCR) for markers identifies emerging NC lineages in vivo (A) Confocal micrographs of isHCR for markers of skeletal and pigment progenitors in PA1 at 24 hpf in *tg(sox10:lynTdTomato)* embryos where NC plasma membranes are marked by tdTomato (red). Expression of *phlda3* (green in upper panels) overlaps with *dlx2a* (magenta) in the ventral portion of the migratory stream, and *atp6ap2* expression (green in lower panels) overlaps with *mitfa* (magenta) in the dorsal portion. (B) Local regression graphs for markers of early NC, pigment, and skeletal progenitors (color code corresponds to Figures 3C and 3D), showing expression levels (y axis) across pseudotime (x axis). Lines indicate moving averages for each branch. Temporal expression profiles for *phlda3* and *sox11a* strongly resemble that of *dlx2a*, while profiles of *phlda1* and *atp6ap2* resemble that of *mitfa*. (C) Confocal micrograph of isHCR for *neurog1* (green), which marks neural progenitors in PA1 at 24 hpf, in *tg(sox10:lynTdTomato)* embryos where NC plasma membranes are marked by tdTomato (gray). (D) Feature plots showing expression of *twist1b* and *twist1a*. First two panels show individual expression patterns (blue and green, respectively). Third panel shows overlapping expression in teal. For micrographs, scale bars represent 50 μm.

Skeletal and pigment lineage markers included many that were expected, such as *dlx2a* and *mitfa,* respectively. However, numerous genes with no previously characterized role in NC were co-expressed in these lineages as well. Among these, *sox11a* and *plextrin homology-like domain a3* (*phlda3*) were expressed specifically in putative skeletal progenitors, while *ATPase H*+ *transporting accessory protein 2* (*atp6ap2*) and *phlda1* were expressed in putative pigment progenitors as early as 18 hpf (Figure S2C). To confirm the specificity of their expression *in vivo*, we performed *in situ* hybridization chain reaction (isHCR). Strikingly, as predicted, at 24 hpf, *phlda3* was co-expressed with *dlx2a* while *atp6ap2* expression overlapped with *mitfa* (Figure 4A). *sox11a* and *phlda1* had similar expression patterns *in vivo* (Figures S4A and S4B). Based on their expression in pseudotime, each of these genes had a temporal expression profile that closely matched canonical skeletal and pigment progenitor markers (Figure 4B). Taken together with their spatial patterns of expression with isHCR, these genes are strong candidates for factors involved in the development of these NC lineages. The neural progenitor branch we identified computationally was confirmed *in vivo* with HCR to detect expression of *neurog1* (Figure 4C). Twist1 is also a well-studied regulator of early NC emigration (Simões-Costa and Bronner, 2015), but interestingly, the emergence of a skeletal progenitor signature in our data also clearly correlated with a shift in expression of two Twist1 orthologs; early NC cells expressed *twist1b*, while later putative skeletal progenitors expressed *twist1a*. Notably, neither pigment nor neural and glial branches expressed either Twist1 ortholog (Figure 4D).

The TF *foxd3* plays numerous roles in NC development, including NC induction as well as specification of the neural and glial lineage (Dottori et al., 2001; Lister et al., 2006; Montero-Balaguer et al., 2006; Stewart et al., 2006). Consistent with this, we observed *foxd3* expression in early NC cells, reduced expression in transitional cells, and later upregulated expression in neural and glial cells in our scRNA-seq dataset (Figures S2A and S2C). To confirm these temporal dynamics of *foxd3* expression *in vivo*, we performed isHCR and found that expression was high at 12 hpf, significantly lower by 18 hpf, and increased again by 24 hpf (Figures S2D and S2E), consistent with our pseudotemporal analysis in scRNA-seq data (Figure S2F). In addition, although changes in expression levels based on scRNA-seq data at 12 and 18 hpf both occurred broadly across all NC cells, the later increase at 24 hpf was restricted to a small subset of cells (Figure S2G). These observations are consistent with previous work (Curran et al., 2010) and the interpretation that *foxd3* shifts from a broad early NC marker to a specific neural and glial lineage specifier, lending strong support that other temporal and lineage-specific features of our scRNA-seq analyses are accurate.

### A distinct gene expression signature defines a transitional population of NC cells mid-migration

**Figure 5.**
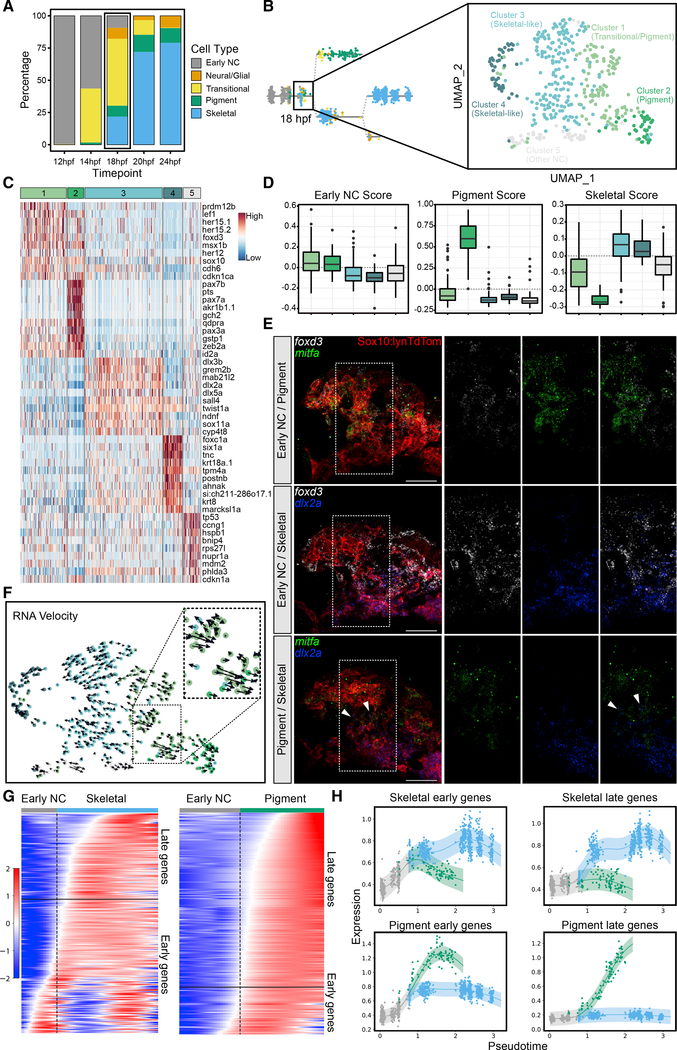
Analysis of 18 hpf scRNA-seq dataset shows emergence of distinct NC lineages as well as transitional cell states (A) Stacked barplot showing percentages of cell types at each time point from 12 to 24 hpf. Color code corresponds to Figure 2C. (B) New larger scRNA-seq dataset generated at 18 hpf. Dimensionality reduction and unbiased clustering revealed 5 distinct clusters: 2 skeletal-like clusters; a pigment cluster; a transitional cluster; and a cluster showing signs of cell stress. Color code distinct from (A). (C) Heatmap showing top 10 marker genes for each cluster; colored bar across the top indicates color code corresponding to (B). (D) Boxplots showing early NC, pigment, and skeletal module scores for each cluster. Color coding corresponds to (B) and (C). (E) Confocal micrographs of isHCR for known markers of early NC (*foxd3*, white), pigment (*mitfa*, green), and skeletal (*dlx2a*, blue) progenitors in *tg(sox10:-lynTdTomato)* embryos where NC plasma membranes are marked by tdTomato (red). Expression of *foxd3* overlaps strongly with *mitfa* and to a lesser extent with *dlx2a*. Although *mitfa* and *dlx2a* mostly do not overlap, a few cells do have overlapping expression (indicated by white arrows). (F) RNA velocity showing differentiation trajectories (arrows) for all cells. The transitional population (inset) is split between progressing toward the skeletal (blue) and pigment (green) clusters. (G) Heatmaps for pigment and skeletal branches with cells ordered by pseudotime. One set of genes marks the transition from early NC to skeletal, and another marks the transition from early NC to pigment. Delineation between early and late genes along these trajectories is marked by horizontal black lines. (H) Local regression graphs showing expression scores for early and late pigment and skeletal markers with cells ordered by pseudotime. A clear branch point marks emergence of pigment (green) and skeletal (blue) lineages. For micrographs, scale bars represent 50 μm.

The analysis of single-cell expression profiles near branchpoints in our pseudotime analyses pinpointed 18 hpf as the stage at which heterogeneity begins to increase in the cranial NC of PA1 in zebrafish (Figure 5A). To gain deeper insights into the apparent rapid developmental changes taking place at 18 hpf, we generated a second, larger scRNA-seq dataset at this stage, containing 420 NC cells. Dimensionality reduction and clustering in Seurat resulted in five clusters with distinct expression profiles (Figure 5B). One cluster showed a clear pigment progenitor expression signature, while two clusters had expression profiles indicative of the skeletal lineage. Another cluster appeared relatively undifferentiated, with low-level expression of both pigment and skeletal lineage markers. The fifth cluster was even less distinct, marked by heat shock and cell cycle genes and possibly stressed and unhealthy cells that were missed initially. However, notably, these cells expressed some distinct factors, including *matrix metalloprotease 2* (*mmp2*), perhaps indicative of a distinct phenotype within the migratory stream (Figures 5B–5D and S4C).

To confirm our scRNA-seq evidence for early NC lineage specification events at 18 hpf *in vivo*, we again performed isHCR for genes thought to mark either a pigment or skeletal progenitor identity. *foxd3* expression was relatively broad across the migratory stream, while both *mitfa* and *dlx2a* marked subsets of NC cells, consistent with the idea that some transitional cells exist at this time point for both lineages. Domains of *mitfa* and *dlx2a* isHCR labeling largely did not overlap in NC cells of the PA1 migratory stream, with *dlx2*+ NC cells largely occupying the ventral region of the stream and *mitfa*+ NC cells restricted to the dorsal region. However, a small number of cells did express both *mitfa* and *dlx2a*, possibly representing cells in the process of fate transition (Figure 5E). We also performed RNA velocity analysis (La Manno et al., 2018), and this confirmed that NC differentiation trajectories within the transitional population largely progressed toward either the pigment or skeletal clusters (Figure 5F). Thus, our *in vivo* isHCR and scRNA-seq data at 18 hpf both indicated the presence of NC cells with pigment and skeletal progenitor signatures, with many cells still in transition. Transitional cells were identified by a distinct expression profile characterized by overlapping expression of early NC markers, like *sox10*, *foxd3*, and *pax3a*, and low-level expression of later lineage markers, including *dlx2a*, *mitfa*, and *sox11a*. Other lineage markers, like *pax7b* and *dlx5a*, were absent and likely only appear later as these subpopulations further differentiate.

In support of the results obtained with RNA velocity, when cells were ordered by pseudotime, a set of genes marking the transition from early NC to skeletal and another marking the transition from early NC to pigment revealed a clear branch in expression profiles (Figures 5G and 5H). We explored this branching event further by identifying differentially expressed genes over time using TradeSeq and ordering them based on their on time. When grouped into early and late genes, depending on expression onset before or after the branch point, we found a stepwise process similar to that described by Soldatov et al. (2019), which was further confirmed in the additional 18-hpf dataset.

### Combined bulk and scRNA-seq reveal Wnt signaling dynamics in developing NC

**Figure 6.**
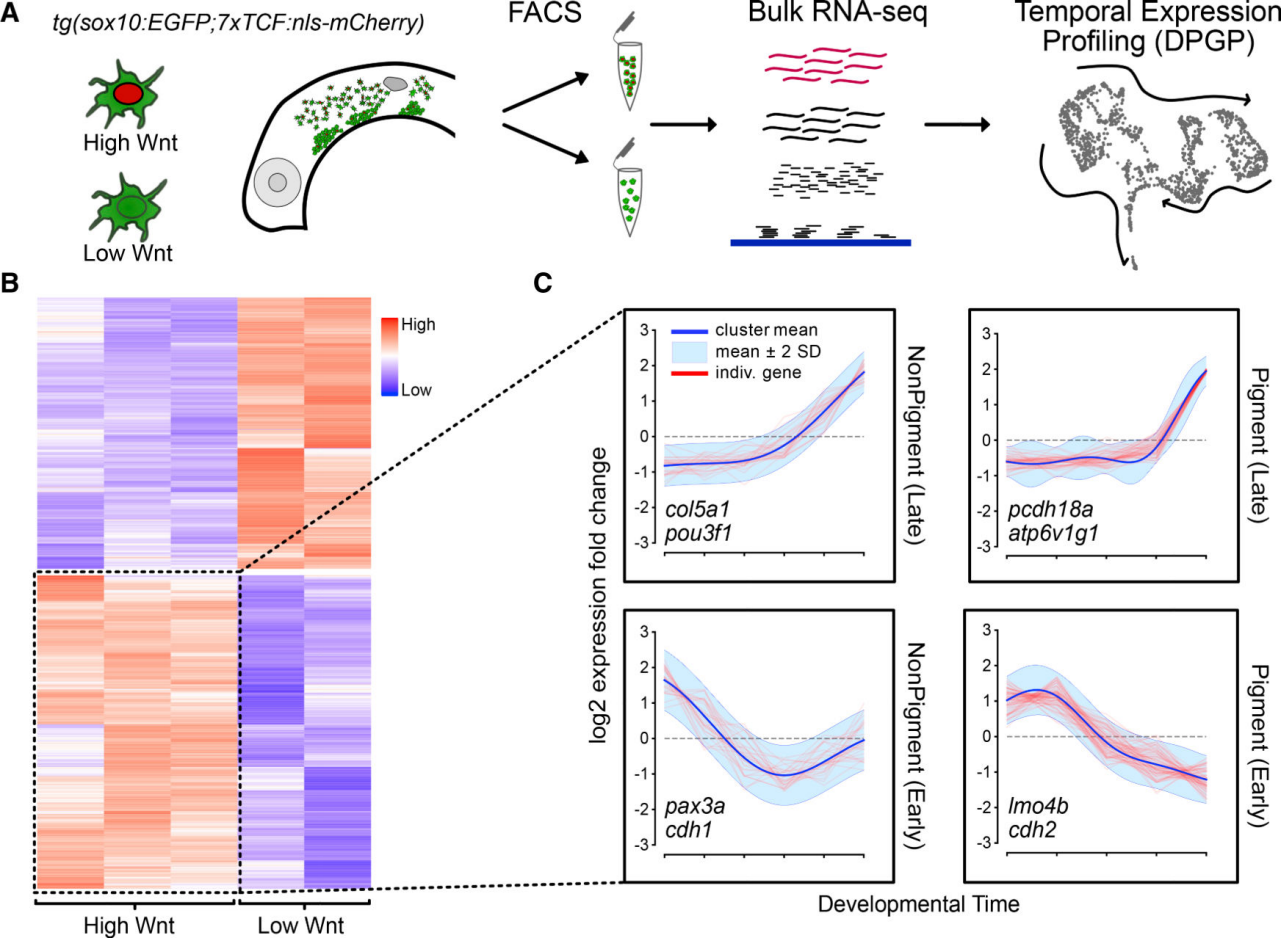
Bulk RNA-seq of a transgenic Wnt reporter combined with scRNA-seq analysis reveals temporal kinetics of Wnt signaling (A) Diagram showing experimental design. Lateral views of the head of a *sox10:EGFP;7xTCF:nls-mCherry* transgenic show migrating NC cells with either high (high Wnt) or low (low Wnt) mCherry levels were isolated via FACS at 24 hpf and then combined for bulk RNA sequencing. Differentially expressed (DE) genes were then analyzed using our scRNA-seq timeline to uncover temporal patterns. (B) Heatmap showing DE genes between high-Wnt and low-Wnt cells. (C) Wnt-upregulated genes were clustered based on their temporal profiles in the scRNA-seq timeline using DPGP (Dirichlet Process Gaussian Process). The timeline (x axis) was split into pigment (right panels) and non-pigment (left panels) branches to separate out Wnt-regulated genes that specifically drive pigment specification. For each branch, two major clusters of genes have either early (lower panels) or late (upper panels) expression signatures. Two genes with these profiles from each temporal cluster are indicated as examples within the plots.

Cell-cell interactions during NC migration likely drive the emergence of the lineage-specific signatures that we observe by scRNA-seq. Many different Wnt ligands and receptors facilitate communication between NC cells and their surrounding tissues during induction, migration, and lineage specification (Ji et al., 2019). To gain insights into the dynamics of Wnt ligand-receptor interactions during cranial NC migration, we generated transcriptomic profiles of Wnt-responsive NC cells using a transgenic line of zebrafish carrying a fluorescent Wnt reporter, tg(*7xTCF:mcherry*), thought to primarily respond to canonical Wnt signaling (Moro et al., 2012). By combining this with tg(*sox10:egfp*) to mark NC cells, at 24 hpf, we FACS sorted double-transgenic NC cells exhibiting high levels of Wnt responsiveness (“high-Wnt,” mCherry+) from those with much lower Wnt responses (“low-Wnt,” mCherry−). Bulk RNA libraries were then constructed and sequenced from the high and low Wnt-responsive NC populations (Figure 6A). 1,917 genes were differentially expressed (DE) between the two populations (Figure 6B). From these DE genes, 1,027 genes were upregulated in the high-Wnt population. Because Wnt signaling has such varied functions across all stages of NC development, we attempted to disentangle the temporal role of each gene in the profile. We utilized our scRNA-seq timeline and the tool DPGP (Dirichlet Process Gaussian Process) (McDowell et al., 2018) to identify shared temporal profiles among groups of genes from the bulk transcriptomic data by gene clustering. Because canonical Wnt signaling promotes pigment cell fates (Curran et al., 2010), we analyzed the pigment and non-pigment branches separately. The combined analyses identified 446 Wnt-responsive genes that were organized into groups corresponding to early and late development in both pigment and non-pigment trajectories (Figure 6C; Table S3). This list was further refined by filtering for genes that overlapped with the GO term for “Wnt signaling pathway” (GO: 0016055) to arrive at a list of putative Wnt targets (Table S4).

Having analyzed Wnt responses in migrating cranial NC cells, we next investigated heterogeneity in the expression of Wnt receptors in NC cells as well as Wnt ligands across all cell types in our FACS-sorted samples. Several Wnt ligands, *wnt4*, *wnt7bb*, *wnt3*, and *wnt8b*, were expressed strongly in cells with a neuroectodermal gene expression profile, while *wnt7aa*, *wnt11*, and *wnt11r* were expressed in cells with an epithelial profile. *wnt11* and *wnt11r* were also expressed in the mesodermal and cardiac subpopulation (Figure S5A). Interestingly, there was a dramatic shift in expression of Wnt receptors in the NC over the time course of migration (Figure S5B). Notably, at 18 hpf expression of a *frizzled 7* (*fzd7a*) highly expressed in early NC cells was reduced, while expression of other genes such as *atp6ap2* was strongly upregulated in cells showing a pigment progenitor gene expression signature. Expression of several other canonical and noncanonical Wnt receptors increased at 20–24 hpf, near the end of NC migration into the mandibular arch, including *ror1*, *fzd3b*, and *fzd2*. The Wnt receptor profile of each NC subtype was even more striking, particularly the pigment-specific expression of *atp6ap2*, skeletal-specific expression of *fzd8a*, and neural- and glial-specific expression of *mcama* (Figure S5B).

We also performed cell-cell communication network analyses on our NC scRNA-seq datasets using the SoptSC package (Wang et al., 2019), which predicts signaling between cells in scRNA-seq data through expression of pathway components. To facilitate this analysis, we generated an unbiased list of directional NC Wnt targets (up or downregulated) by filtering DE genes from our bulk Wnt-reporter RNA-seq data using the “Wnt signaling” GO term. With the full set of ligands, receptors, and Wnt-regulated genes expressed in our scRNA-seq datasets, we calculated signaling probabilities between each NC cell subtype and surrounding tissues at 14, 18, 20, and 24 hpf. The resulting signaling probabilities suggest changes in the sources of signals as well as their strength over time (Figure S5C). Surprisingly, we also found evidence for Wnt-dependent communication between NC cells, weak at 14 hpf but stronger at 24 hpf.

**Figure 7.**
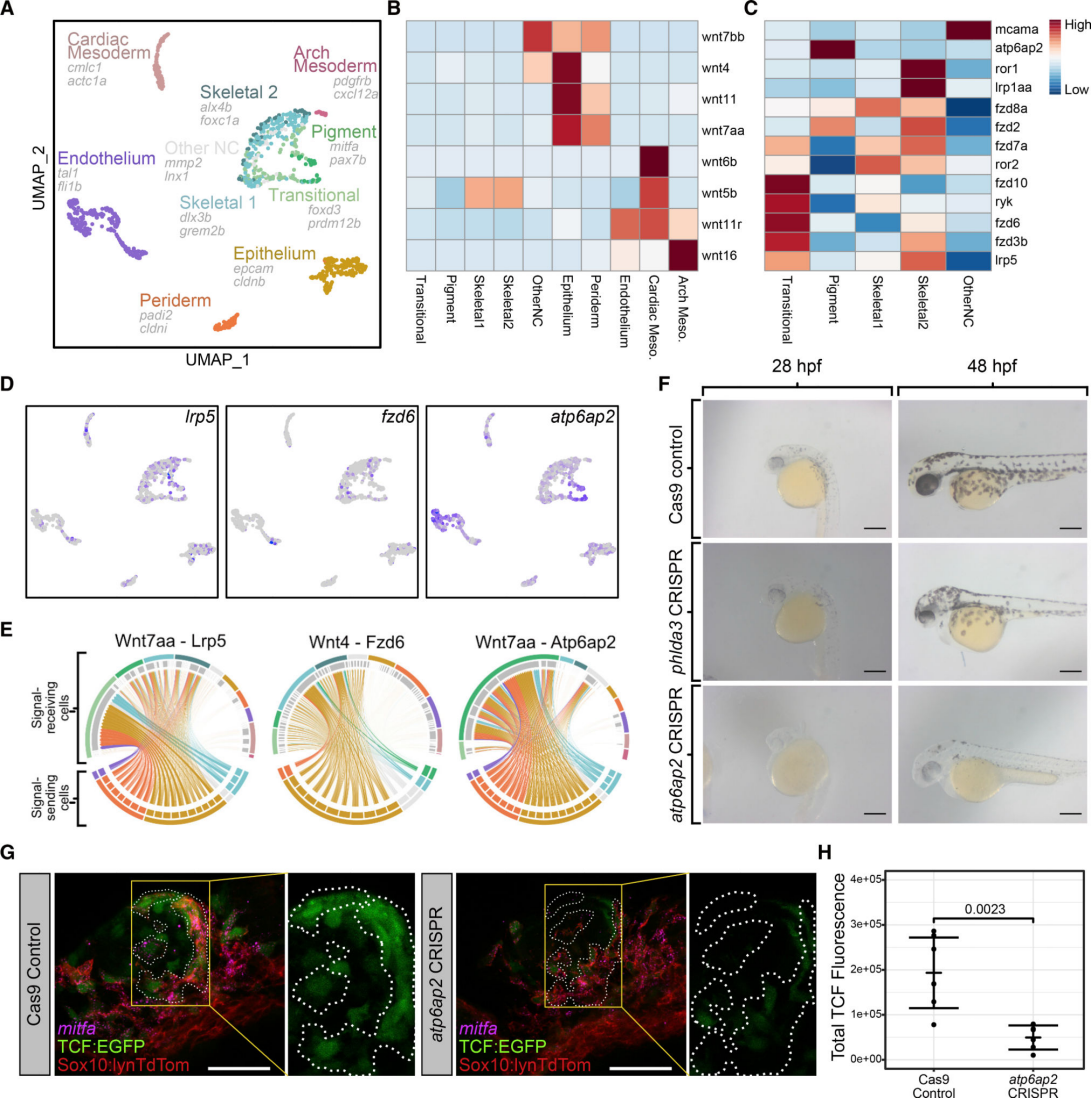
Emergence of pigment and skeletal lineages in NC cells coincides with Wnt signals from epithelial tissues (A) UMAP showing all cell types isolated from photoconverted PA1 NC cells. Cell types determined from top DE genes and combined with NC subtype identities from our NC-specific analysis. (B) Heatmap showing average expression of Wnt ligands in each cell type. (C) Heatmap showing average expression of Wnt receptors in each cell type. (D) Feature plots showing expression of Wnt receptors *lrp5*, *fzd6*, and *atp6ap2*. (E) Circos plots showing imputed signaling interactions. Top segments of each circle indicate signal-receiving cells. Bottom segments indicate signal-sending cells. Each line indicates a signaling event between two individual cells. Lines are colored according to the cell type identity of the signal-sending cell. Colors are consistent with UMAP. (F) Live embryos injected with either Cas9 alone (top row) or Cas9 and 4 gRNAs targeting *phlda3* (middle row) or *atp6ap2* (bottom row) shown in lateral views at 28 hpf (left column) and 48 hpf (right column). Note reduced black melanocyte pigmentation with *atp6ap2* CRISPR. (G) Confocal micrographs showing Wnt-responsive TCF-EGFP (green, outlined by dashed white lines) and *mitfa* (magenta) expression in PA1 at 24 hpf in *tg(sox10:lynTdTomato;7xTCF:EGFP)* embryos where NC plasma membranes are marked by tdTomato (red). (H) Quantification of 7xTCF:EGFP using corrected total fluorescence intensity between control embryos (n = 7 embryos; mean = 193,407) and *atp6ap2* CRISPR embryos (n = 6 embryos; mean = 49,567). Wilcoxon rank-sum test p = 0.0023. Dots represent mean in individual embryos. Lines represent mean within conditions. Error bars represent mean ± SD. For micrographs, scale bars represent 50 μm.

Because initial specification of at least some lineages appears to be taking place by 18 hpf, we investigated Wnt signaling dynamics at this stage in our larger 18-hpf dataset. Analysis of all cells revealed several non-NC cell types expressing Wnt ligands, including cardiac mesoderm, endothelium, and epithelium as well as a small arch mesodermal population and a periderm population (Figure 7A). Wnt ligands had uneven distribution across cell types. *wnt6b*, *wnt11r*, and *wnt16* were mostly expressed in the mesodermal populations, while *wnt7bb*, *wnt4*, *wnt11*, and *wnt7aa* were enriched in epithelial and peridermal cells (Figure 7B). Wnt receptors also had distinct expression patterns across NC cell subtypes. Several receptors, including *fz6* and *lrp5*, were expressed strongly in the transitional population but also in pigment and skeletal cells. Pigment cells again had very strong enrichment of *atp6ap2* (Figures 7C and 7D). SoptSC analyses at 18 hpf revealed three notable lineage-specific interactions: signaling from epithelial and peridermal cells to transitional NC cells via *wnt7aa* and *lrp5*; signaling from epithelial cells to skeletal NC lineages via *wnt4* and *fzd6*; and signaling from epithelial and peridermal cells to NC-derived pigment progenitors via *wnt7aa* and *atp6ap2* (Figure 7E).

### Requirements for atp6ap2 in pigment cell development

The *atp6ap2* gene encodes a component of the v-ATPase complex, which also functions as an intracellular renin receptor and Wnt co-receptor (Cruciat et al., 2010; Ichihara and Yatabe, 2019). Although mutants and knockdowns of *atp6ap2* in zebrafish have been reported to cause biliary defects (EauClaire et al., 2012), it has not been implicated in NC development. Consistent with our scRNA-seq data, expression in migrating cranial NC was clear at 24 hpf (Figure 4A). To test *atp6ap2* function, we generated targeted mutations in zebrafish using multiplexed CRISPR-Cas9 genome editing (Wu et al., 2018). F0 embryos injected with gRNAs targeting *atp6ap2* (hereafter called “crispants”) displayed a severe loss of pigmentation compared to siblings injected with Cas9 alone or Cas9 and gRNAs targeting the skeletal marker *phlda3* (Figure 7F). Melanocytes were both reduced in number and size in embryos at 48 hpf, possibly due to defects in their specification. To test this hypothesis, we examined *mitfa* expression by isHCR but observed no differences in *mitfa* expression between control embryos and *atp6ap2* crispants (Figures S6A and S6B), suggesting that defects are in later differentiation or survival. Because *atp6ap2* may act as a Wnt co-receptor, we next investigated whether Wnt signaling was affected in *atp6ap2* crispants using tg(*7xTCF:EGFP*). Crispants showed a marked decrease in EGFP intensity in *mitfa*+ cells, as well as in other Wnt-responsive regions (Figures 7G and 7H). Together, these results indicate a previously unknown role for *atp6ap2* in NC and maintenance of melanocytes through canonical Wnt signaling and demonstrate the utility of predictive signaling analysis using single-cell data in identifying regulatory genes.

## DISCUSSION

NC development involves coordinated fate decisions as cells are migrating to achieve proper formation of a huge variety of cell types. Previous single-cell studies have reported expression profiles of cranial NC lineages but lack detailed spatial information and rely on pseudotime to derive transitional cell states. In this study, we present a single-cell transcriptomic timeline of zebrafish cranial NC development with cells isolated from the migratory stream that contributes to PA1 at closely spaced time points during their migration. Thus, the data derive from a known NC subpopulation and capture real-time gene expression dynamics to contextualize inferred lineage connections and clarify the temporal transitions that cranial NC cells undergo as they differentiate. By assimilating multiple inputs and outputs in the NC gene-regulatory network using several computational pipelines, we identify a key transitional cell stage mid-migration when heterogeneity in gene expression arises and subpopulations of skeletal and pigment progenitors begin to emerge, followed by neural and glial progenitors. We uncover several genes that strongly mark these lineage bifurcations. In addition, through integration of bulk RNA-seq from a Wnt reporter transgenic with our single-cell data, we model lineage-specific Wnt signaling dynamics and with targeted genome editing provide data to suggest an important function for the Wnt-regulatory factor, Atp6ap2, in NC-derived pigment cell development. Together, our results reveal transcriptional changes and cell-cell signals that coordinate NC cell migration and fate.

### Transcriptional biases toward pigment, skeletal, and glial lineages arise mid-migration in NC cells that populate PA1

Previous lineage-tracing studies in a variety of species have provided evidence both for multipotency and lineage restrictions in premigratory NC cells (Baggiolini et al., 2015; Krispin et al., 2010; McKinney et al., 2013; Schilling and Kimmel, 1994). Our scRNA-seq analyses found no evidence for heterogeneity in the gene expression signatures of premigratory NC cells destined for PA1 in zebrafish. These results suggest either that premigratory NC cells are entirely unspecified toward any particular lineage or that lineage specification is not detectable at the level of transcription prior to migration. The first signs of transcriptional heterogeneity become apparent at 18 hpf, when subsets of cells express multiple markers of pigment and skeletal precursors. Notably, these two sets of precursors are already spatially segregated in the NC of PA1 at this stage (Figure 3). *mitfa*+ pigment progenitors are restricted to the dorsal region of the migratory stream, whereas *dlx2a*+ skeletal progenitors occupy the ventral region. NC cells with signatures of glial precursors are detected slightly later in migration, at 20 hpf. This demonstrates that lineage decisions and spatial organization are coordinated early in cranial NC migration. This could be explained by the predicted changes in Wnt signaling that we have observed, as well as other signals between NC cells and their environment. Investigation into the spatial distribution of signaling molecule expression *in vivo* would help to elucidate the mechanics governing this apparent coordination.

The emergence of the pigment lineage followed by later specification of glial lineages is somewhat surprising, as the prevailing model describes a bifurcation between these two lineages occurring simultaneously as the result of competing interactions between *foxd3* and *mitfa* (Curran et al., 2010). Our results support this transitional overlapping expression of *mitfa* and *foxd3*, probably representing the interplay whereby *mitfa* expression inhibits *foxd3* as the pigment lineage is specified (Figure 3). However, it seems the glial lineage decision may be driven by a later re-deployment of *foxd3* in a population of NC cells distinct from this *foxd3+*/*mitfa*+ transitional pigment population (Figures 2 and S2). These findings clarify the timing of cranial NC lineage decisions, at least in PA1, and provide context to the bifurcation between pigment and glial cell fates.

Our findings are somewhat in disagreement with previously published single-cell transcriptomics studies of the NC, which arrived at varying interpretations regarding the temporal aspects of lineage specification. scRNA-seq data in chick cranial NC identified a putative “neural” NC population prior to migration characterized by low expression of NC specifiers *TFAP2A/B* and *FoxD3* and high levels of *Sox3* (Williams et al., 2019). We found no evidence of an early neural NC progenitor population but did see a similar expression profile in NT cells that we captured (Figure S1). Our findings also differ from scRNA-seq results in mice, which revealed an early bifurcation between neural and mesenchymal lineages in NC cells isolated from the head-trunk boundary with no obvious specification of pigment cells (Soldatov et al., 2019). In addition to a different anterior-posterior subpopulation, this may also reflect differences in the development of larval zebrafish and early embryonic mouse melanocytes, the latter of which largely derive from Schwann cell precursors (Adameyko et al., 2009). These discrepancies could reflect our computational integration of the temporal data, e.g., parallel analyses of pseudotime using Monocle 3 and PAGA combined with real-time data. Alternatively, they could reflect real regional differences in lineage specification among NC subpopulations (different migratory streams) or differences between species. A combinatorial analysis of these currently available single-cell NC datasets with the added context of our temporal single-cell data could help to reconcile these discrepancies and build a more robust understanding of early lineage specification.

### Deployment of Twist1 paralogs in zebrafish NC development

One striking marker of the skeletogenic lineage in our data is *twist1a*. Twist1 functions as both a NC specifier and regulator of mesenchymal NC cell lineages (Simões-Costa and Bronner, 2015). In mammals and amphibians, early Twist1 expression in the neuroepithelium marks cells that will undergo EMT and give rise to NC, with expression persisting in NC cells that later progress toward skeletal fates (Hopwood et al., 1989; Ishii et al., 2003; Soo et al., 2002). Zebrafish have two Twist1 orthologs, *twist1a* and *twist1b*, which likely arose with the whole-genome duplication in teleosts and are often grouped together in the context of NC development (Germanguz et al., 2007). Here, we show that they have striking mutually exclusive temporal expression profiles. Although *twist1b* strongly marks early NC cells, its expression quickly drops off within the first 6 h of migration, during which time NC cells with a signature of skeletal progenitors upregulate *twist1a* expression. This strongly suggests that, in zebrafish, the *twist1* duplicates diverged in function during evolution, with *twist1b* retaining one ancestral function as a NC specifier and *twist1a* another function in driving migration in skeletal lineages, consistent with the gene duplication and divergence hypothesis (Force et al., 1999). This serves to highlight species-specific strategies in NC development.

### Distinct Wnt signaling components correlate with NC heterogeneity and a role for Atp6ap2

Our computational analyses of Wnt signaling synthesize input from bulk and scRNA-seq data, allowing us to predict interesting trends in individual gene expression trajectories and cell-cell communication. This reveals evidence for several lineage- and stage-specific interactions, including (1) epithelial Wnt7aa signaling to Lrp5 in transitional NC cells, (2) epithelial Wnt4 signaling to skeletal progenitors via Fzd6, and (3) epithelial Wnt7aa signaling to pigment progenitors via Atp6ap2 (Figure 6E). Wnt signaling has been implicated in multiple aspects of NC induction, delamination, and migration, as well as multipotency and cell fate determination (reviewed in Ji et al., 2019 and Rocha et al., 2020), including several of the components identified here (Willems et al., 2015). These results highlight the dynamic nature of Wnt signaling in the NC. Further investigation into individual lineage- and stage-specific Wnt signals presents an exciting opportunity to uncover mechanisms driving NC lineage specification.

As evidence of the promise of such an approach, our marker gene and predictive signaling analyses unbiasedly identified *atp6ap2* as a marker and putative regulator of pigment progenitor development potentially through canonical Wnt signaling. This gene encodes the (pro)renin receptor protein ((P)RR), which has numerous cellular functions. (P)RR is a receptor that binds prorenin to activate tissue RAS, but it is also a subunit of the v-ATPase complex and is vital for proper vesicular acidification in the kidney and heart (Ichihara and Yatabe, 2019). Atp6ap2/(P)RR also serves as a co-receptor for LRP6 in canonical Wnt signaling during anterior-posterior patterning of the central nervous system (CNS), linking LRP6 to the v-ATPase complex to activate the Wnt-β-catenin pathway upon binding of Wnt ligand (Cruciat et al., 2010). Here, we demonstrate that *atp6ap2* expression marks NC cells that adopt pigment cell fates and is vital for their survival and maturation. Further, we show that this defect correlates with a reduction in canonical Wnt signaling, supporting the notion that the function of Atp6ap2 in melanocytes resembles its previously identified role in mediating canonical Wnt signaling in the CNS. The loss of pigmentation we observe in *atp6ap2* crispants is consistent with a zebrafish mutant for *atp6ap2* called *pekin*, which exhibits biliary defects as well as hypopigmentation, although the mechanism underlying this latter phenotype has not been investigated (EauClaire et al., 2012). Wnt signaling is required both for specification of the pigment lineage and for later differentiation of mature melanocytes (Vibert et al., 2017). We previously characterized a role for the v-ATPase-associated proteins Rbc3a and Atp6v0a1 in controlling migration of pigment progenitor NC cells through modulation of canonical Wnt signaling (Tuttle et al., 2014). This role for Atp6ap2 in pigment cell development post-migration further highlights the multifaceted importance of v-ATPase-mediated canonical Wnt signaling.

### Limitations of the study

This study is limited somewhat by the fact that each time point (aside from 18 hpf) in the timeline is represented by a single 10x library. Therefore, although the emergence of lineages has been validated through *in vivo* staining, some of the broad timepoint-specific expression differences could be technical in nature.

## STAR★METHODS

### RESOURCE AVAILABILITY

#### Lead contact

Further inquiries and and requests for resources or materials should be directed to the Lead Contact, Dr. Thomas F. Schilling (tschilli@uci.edu).

#### Materials availability

Transgenic lines, HCR probes, and gRNA primers will be made available upon request.

#### Data and code availability

- Bulk RNA-seq and single-cell RNA-seq data have been deposited to the Gene Expression Omnibus (GEO) and are publicly available as of the date of publication. Accession numbers are listed in the Key resources table. Microscopy data will be shared by the lead contact upon request.
- Custom code written for this project has been deposited on GitHub and is publicly available. The DOI and URL for the GitHub repository are listed in the Key resources table.
- Any additional information required to reanalyze the data reported in this work paper is available from the lead contact upon request.

### EXPERIMENTAL MODEL AND SUBJECT DETAILS

#### Zebrafish

Wild-type or transgenic zebrafish of the AB strain were used for all of the experiments. Transgenic lines used in this study include Tg(*sox10:nEOS*)^w18^ (Curran et al., 2010), Tg(*sox10:lyn-tdTomato*)^ir1040^ *Tg(*−*7.2sox10:EGFP)*^ir937^ (Schilling et al., 2010), Tg(*7XTCF:EGFP*)^ia4^ (Moro et al., 2012), and Tg(*7XTCF:nls-mCherry*)^ia5^ (Moro et al., 2012). All zebrafish lines were maintained according to standard protocols (Westerfield, 2000). Embryos between 12–24 hpf were obtained from natural breedings and staged as described in Kimmel et al. (1995). For NC cell dissociation and FACS sorting, cells were either photoconverted in transgenic Tg(*sox10:nEOS*) zebrafish embryos or double labeled using two-color combinations of Tg(*sox10:lyn-tdTomato*), *Tg(*−*7.2sox10:EGFP)*, Tg(*7XTCF:EGFP*) and Tg(*7XTCF:nls-mCherry*). Procedures involving animals were approved by the Institutional Animal Care and Use Committee at the University of California, Irvine.

### METHOD DETAILS

#### Embryo dissociation and FACS

Transgenic Tg(*sox10:nEOS*) zebrafish embryos were mounted in 1% agarose and imaged on a Nikon C1 confocal using a 20x objective. NC cells of the PA1 NC migratory stream were converted by drawing a region of interest (ROI) around the targeted cells and then exposing the ROI to 405 laser light for 10 seconds. Embryos were then dissociated by light mechanical disruption using a p1000 pipette and trypsin/collagenase P incubation for 15 minutes as described in Barske et al. (2016). Dissociated cell samples were processed on a BD FACS Aria II cell sorter. Photoconverted (red) cells were separated from unconverted (green) cells based on intensity of red/green fluorescence. For Wnt reporter experiments, *TCF:mCherry; Sox10:LynEGFP* double positive cells and *Sox10:LynEGFP*+*;TCF:mCherry*− cells were FACS sorted.

#### 10x library construction and sequencing

Single cell suspensions were processed on a 10x Chromium platform for single-cell library construction. Libraries were then sequenced on a HiSeq2500 (Illumina).

#### Read mapping and pre-processing

FASTQ files were mapped to the zebrafish transcriptome GRCz11 using CellRanger. Mapped reads for the 12–30 hpf timeline were aggregated using the CellRanger pipeline before further computational analysis. Counts matrix normalization and scaling were performed using Seurat v3 (Stuart et al., 2019) using the NormalizeData and ScaleData functions.

#### Bulk RNA-seq library construction and sequencing

RNA was extracted from cell lysates using the RNEasy Micro Kit (QIAGEN). cDNA libraries were generated following the Smart-seq2 protocol (Picelli et al., 2014). Libraries were then sequenced on a NovaSeq6000 (Illumina) at a depth of ~20M reads per sample.

#### Multiplex CRISPR-Cas9 genome editing

Perturbations of *atp6ap2* were achieved using the multiplex CRISPR technique described by Wu et al. (2018). PCR was carried out with 4 unique primers containing a T7 promoter and guide sequence and a scaffolding primer to generate templates for gRNA synthesis. Four distinct gRNAs targeting the coding region of *atp6ap2* were then synthesized from these templates using T7 MegaShortScript kit (Ambion). A solution of 150 ng/μl of each gRNA and 5μM Cas9 protein (IDT) was incubated at 37°C for 5 minutes. This solution was injected along with 0.5% phenol red into 1-cell stage embryos at a volume of 1 nl/embryo. gRNA primer sequences were taken from the database provided by Wu et al. (2018) and can be found in the Key resources table.

#### *In situ* hybridization chain reaction

HCR probes were ordered from and designed by Molecular Technologies (Los Angeles, CA). Whole mount HCR was carried out as described by Choi et al. (2014). Briefly, embryos were fixed in 4% paraformaldehyde overnight at 4°C, dehydrated with 100% methanol overnight at −20°C, and then rehydrated through a series of washes with 3:1, 1:1, and 1:3 solutions of methanol/1XPBS. Embryos were then prehybridized with 30% probe hybridization buffer at 37°C for 30 minutes and then incubated with 2 pmol of each probe in 30% probe hybridization buffer overnight at 37°C. Embryos were washed and then incubated for 30 minutes at room temperature in the dark with 30 pmols of amplification hairpins. NCBI Accession numbers used to design each probe can be found in the Key resources table.

### QUANTIFICATION AND STATISTICAL ANALYSIS

#### Quantitative analyses of HCR and Wnt reporter

Raw fluorescence intensities were measured by drawing ROIs in ImageJ, and corrected total cell fluorescence (CTCF) values were calculated using the formula CTCF = Integrated Density − (Area × Mean background fluorescence). Mean intensity values between time points and/or conditions were compared using Wilcoxon signed-rank test. For *foxd3* measurements, means were compared using Kruskal-Wallis ANOVA and then pairwise comparisons were performed using Wilcoxon signed-rank tests. Statistical tests were carried out using R, and plots were generated in R using the ggplot2 package. Other R packages used include: plyr, dplyr, reshape2, and ggpubr. Sample sizes and replicate numbers can be found in the figure legends for each individual analysis. Individual cutoffs for statistical significance are also listed in the figure legends.

#### Bulk RNA-seq data processing and analysis

Reads were mapped to zebrafish genome version GRCz11 and quantified using STAR v2.5.2a and RSEM v1.2.31. Differential expression testing was performed in R using the edgeR package. *TCF:mCherry*+ NC cells were compared to *TCF:mCherry*− NC cells using the edgeR GLM framework with default parameters and false discovery rate (FDR) p value correction. FDR < 0.05 was used as a cutoff for significant differences in expression level. Heatmaps were generated using the ComplexHeatmap R package.

#### Computational analysis of single-cell RNA-seq data

Variable features identification, principal component analysis (PCA), UMAP reduction, integration, and unbiased clustering were performed in Seurat v3 using the FindVariableFeatures, RunPCA, RunUMAP, FindNeighbors, and FindClusters functions. For dimensionality reduction and unbiased clustering, 13 PCs were used in the whole arch analysis, 5 PCs were used in the subclustered NC analysis, and 11 PCs were used in the separate 18 hpf NC analysis. Differential expression analysis in Seurat v3 was used to identify cluster markers by Wilcoxon rank sum test. Cluster markers were then used to manually assign cell types. Visualizations were generated using Seurat v3 and ggplot2 with color palettes from the RColorBrewer and Viridis packages. A subset counts matrix containing TFs was analyzed with the learn_graph function in Monocle3 for trajectory and pseudotime analysis with default parameters. Dimensionality reduction was performed as described above in Seurat v3 using 7 PCs. UMAP embedding was then imported into Monocle3.Identification of temporally distinct TF modules was performed using the graph_test and find_gene_modules functions in Monocle3.

#### Clustering of DE genes via Dirichlet process Gaussian process (DPGP) modeling

For temporal profiling of DE genes from Wnt reporter bulk RNA-seq, genes were clustered using scRNA-seq data for cells from either the pigment branch or the non-pigment branch separately with DPGP (McDowell et al., 2018). Briefly, the DPGP model assumes that the temporal expression of a gene is generated from a mixture of Gaussian processes, such that each Gaussian process corresponds to a gene expression cluster, the parameters of which are further generated by a Dirichlet process. The concentration parameter α for the Dirichlet process was set to 0.2. To obtain temporal expression patterns for each gene, average counts of all cells in a branch for all time points were calculated (except 30 hpf for the pigment branch), which constituted a time series for that gene. DPGP was allowed to infer the optimal clustering that attained maximum *a posteriori* (MAP) likelihood, given those time series of average gene expression.

#### PAGA dimensionality reduction and trajectory inference

The PAGA_tree (Wolf et al., 2019) wrapper in the Dyno (Saelens et al., 2019) package was used to infer developmental trajectories. The dataset was first processed with the default option, recipe_Zheng17 in Scanpy. The method PAGA_tree was then applied with the parameters, n_comps = 50, n_neighbors = 15, resolution = 1, n_dcs = 10. “AAACCTGAGTGGCACA” in the 12 hpf dataset was chosen as the root cell. A simplified model was obtained using Dyno based on which pseudotime was also computed in Dyno. PAGA coarse graph was used to initialize single-cell embedding in ForceAtlas2 (Jacomy et al., 2014). To evaluate robustness of the inferred trajectory we carried out this analysis with 27 different combinations of key parameters, namely, the number of principal components, number of neighbors, and number of variable genes.

#### RNA velocity and TradeSeq

RNA velocity for 18 hpf was computed using the scVelo package (Bergen et al., 2020) using default parameters. We also identified temporal differentially expressed genes using tradeSeq (Van den Berge et al., 2020), ordering and grouping them into early and late genes relative to branch points based on their expression onset. The fitGAM function in tradeSeq package was used to model the association between pseudotime and gene expression along each branch. Specifically, a generalized additive model was used to model the dependencies of gene expression levels on the pseudotime. Wald test were used to determine the differential expression of genes. The top 3000 highly variable genes were used in tradeSeq analysis. The parameter nknot in tradeSeq was set to 6. The DE genes with a p value less than or equal to 0.05 were kept.

#### Probabilistic cell type assignment

Cell Types were assigned based on known markers and TFs derived from TF pseudotemporal analysis (Figure S1) in a supervised approach using the package CellAssign (Zhang et al., 2019). The exact marker genes are listed in the individual csv files. The function cellassign was used with learning_rate = 0.02, shrinkage = TRUE, and a size factor estimated using the computeSumFactors function in scran package (Lun et al., 2016).

#### Cell-cell communication analysis in scRNA-seq data

Communication between cells was predicted from our single-cell RNA-seq data using the SoptSC suite of computational tools (Wang et al., 2019). Briefly, the probability of communication between individual cells was calculated for individual ligand-receptor pairs based on their expression of these factors and expression of downstream target genes. Analysis was performed in R using the R package for SoptSC. The source code for this package is deposited on GitHub and is publicly available. The URL for the GitHub repository is listed in the Key resources table.

#### Inter and Intra-module correlation analysis

Specificity of lineage gene expression modules over developmental time was analyzed using an approach similar to that previously implemented in studies of mouse spatiotemporal NC transcriptomics (Soldatov et al., 2019). Expression scores were generated for each cell using the AddModuleScore function in Seurat v3, taking the lineage markers from Table S2 as input. Pearson correlations were then calculated between each gene on this list and each of the 3 major lineage scores (pigment, skeletal, and neural/glial) across all cells within each time point. Custom code used for this analysis is available at the GitHub URL listed in the Key resources table.

## Supplementary Material

1

2

3

4

5

6

## Figures and Tables

**KEY RESOURCES TABLE:** 

REAGENT or RESOURCE	SOURCE	IDENTIFIER

Chemicals, peptides, and recombinant proteins		

Collagenase P		N/A
Alt-R S.p. Cas9 Nuclease V3	IDT	Cat# 72672

Critical commercial assays		

RNEasy Micro kit	QIAGEN	Cat# 74004
Qubit dsDNA HS assay kit	Invitrogen	Cat# Q32854
Qubit 2.0 Fluorometer	Invitrogen	Cat# Q32866
Tapestation High sensitivity D1000	Agilent	Cat# 5067–5584
SuperScript II Reverse Transcriptase	Invitrogen	Cat# 18064–014
KAPA HiFi Hot Start PCR Ready Mix	KAPA Biosystems	Cat# KK2601
Agencourt Ampure XP Beads	Beckman Coulter	Cat# 63881
Betaine	Sigma Aldrich	Cat# 61962
Chromium Single Cell 3′ Library & Gel Bead Kit v2	10X Genomics	Cat# 120237
Chromium single cell A chip kit, 48 rxns	10X Genomics	Cat# 120236
Chromium Single Cell 3′ GEM, Library & Gel Bead Kit v3, 16 rxns	10X Genomics	Cat# 1000075
Chromium Chip B Single Cell Kit, 48 rxns	10X Genomics	Cat# 1000073

Deposited data		

Raw and analyzed single-cell RNA-seq data	This paper	GEO: GSE168133
Raw and analyzed bulk RNA-seq data	This paper	GEO: GSE168131

Experimental models: organisms/strains		

Tg(−4.9sox10:nEOS)w18	Raible Lab / Curran et al., 2010	ZFIN ID: ZDB-FISH-150901–16731
Tg(−4.9sox10:lyn-tdTomato)ir1040	Our lab / (Schilling et al., 2010)	ZFIN ID: ZDB-ALT-120418–23
Tg(7XTCF:nls-mCherry)ia5	Argenton Lab / Moro et al., 2012	ZFIN ID: ZDB-ALT-110113–2
Tg(−7.2sox10:EGFP)ir937	Our lab / (Schilling et al., 2010)	ZFIN ID: ZDB-ALT-080228–1

Oligonucleotides		

Atp6ap2 CRISPR gRNA primer 1: 5′TAATACGACTCACTATAGGGATCTTTTCACCCGATATGTTTTAGAGCTAGAAATAGC3′	IDT/Wu et al., 2018	N/A
Atp6ap2 CRISPR gRNA primer 2: 5′TAATACGACTCACTATAGGCGCTCTAATCGTTGTGCGGTTTTAGAGCTAGAAATAGC3′	IDT/Wu et al., 2018	N/A
Atp6ap2 CRISPR gRNA primer 3: 5′TAATACGACTCACTATAGGTGGTCATACCTCTTCACTGTTTTAGAGCTAGAAATAGC3′	IDT/Wu et al., 2018	N/A
Atp6ap2 CRISPR gRNA primer 4: 5′TAATACGACTCACTATAGGTGTTCCTCTCTGAGGTTCGTTTTAGAGCTAGAAATAGC3′	IDT/Wu et al., 2018	N/A
gRNA scaffold primer: 5′AAAAGCACCGACTCGGTGCCACTTTTTCAAGTTGATAACGGACTAGCCTTATTTTAACTTGCTATTTCTAGCTCTAAAAC3′	IDT/Wu et al., 2018	N/A
Mitfa HCR probe set	Molecular, Technologies	NM_130923.2
Dlx2a HCR probe set	Molecular, Technologies	NM_131311.2
Foxd3 HCR probe set	Molecular, Technologies	NM_131290.2
Atp6ap2 HCR probe set	Molecular, Technologies	NM_213023.2
Phldal HCR probe set	Molecular, Technologies	NM_001006011.1
Phlda3 HCR probe set	Molecular, Technologies	NM_001002455.1
Sox11a HCR probe set	Molecular, Technologies	NM_131336.1

Software and algorithms		

STAR	N/A	https://github.com/alexdobin/STAR
RSEM	N/A	https://github.com/deweylab/RSEM
edgeR	Robinson et al., 2010	https://bioconductor.org/packages/release/bioc/html/edgeR.html
Seurat v3	Stuart et al., 2019	https://satijalab.org/seurat/index.html
Monocle 3	Cao et al., 2019; Trapnell et al., 2014	https://github.com/cole-trapnell-lab/monocle3
SoptSC	Wang et al., 2019	https://github.com/mkarikom/RSoptSC
DPGP	McDowell et al., 2018	https://github.com/PrincetonUniversity/DP_GP_cluster
PAGA	Wolf et al., 2019	https://github.com/theislab/paga
ForceAtlas2	Jacomy et al., 2014	https://github.com/bhargavchippada/forceatlas2
CellAssign	Zhang et al., 2019	https://github.com/Irrationone/cellassign
Dyno	Saelens et al., 2019	https://github.com/dynverse/dyno
Custom Code for analysis	This Paper	https://github.com/tschilling-lab/, https://doi.org/10.5281/zenodo.5648293
CellRanger	10x Genomics	https://github.com/10XGenomics/cellranger
tradeSeq	Van den Berge et al., 2020	https://github.com/statOmics/tradeSeq
scVelo	Bergen et al., 2020	https://github.com/theislab/scvelo
scran	Lun et al., 2016	https://github.com/MarioniLab/scran

## Highlights

- We provide a single-cell RNA-seq timeline of cranial NC development in zebrafish
- NC cells show no transcriptional signs of lineage specification prior to migration
- Pigment and skeletal fates arise mid-migration and are spatially segregated
- Changes in Wnt signaling across migration correlate with lineage specification

## References

[R1] Adameyko I, Lallemend F, Aquino JB, Pereira JA, Topilko P, Müller T, Fritz N, Beljajeva A, Mochii M, Liste I, (2009). Schwann cell precursors from nerve innervation are a cellular origin of melanocytes in skin. Cell 139, 366–379. 19837037 10.1016/j.cell.2009.07.049

[R2] Baggiolini A, Varum S, Mateos JM, Bettosini D, John N, Bonalli M, Ziegler U, Dimou L, Clevers H, Furrer R, and Sommer L (2015). Premigratory and migratory neural crest cells are multipotent in vivo. Cell Stem Cell 16, 314–322. 25748934 10.1016/j.stem.2015.02.017

[R3] Barske L, Askary A, Zuniga E, Balczerski B, Bump P, Nichols JT, and Crump JG (2016). Competition between Jagged-Notch and Endothelin1 signaling selectively restricts cartilage formation in the zebrafish upper face. PLoS Genet. 12, e1005967. 27058748 10.1371/journal.pgen.1005967 PMC4825933

[R4] Bergen V, Lange M, Peidli S, Wolf FA, and Theis FJ (2020). Generalizing RNA velocity to transient cell states through dynamical modeling. Nat. Biotechnol. 38, 1408–1414. 32747759 10.1038/s41587-020-0591-3

[R5] Bronner-Fraser M, and Fraser SE (1988). Cell lineage analysis reveals multipotency of some avian neural crest cells. Nature 335, 161–164. 2457813 10.1038/335161a0

[R6] Bronner-Fraser M, Sieber-Blum M, and Cohen AM (1980). Clonal analysis of the avian neural crest: migration and maturation of mixed neural crest clones injected into host chicken embryos. J. Comp. Neurol. 193, 423–434. 7440776 10.1002/cne.901930209

[R7] Cao J, Spielmann M, Qiu X, Huang X, Ibrahim DM, Hill AJ, Zhang F, Mundlos S, Christiansen L, Steemers FJ, (2019). The single-cell transcriptional landscape of mammalian organogenesis. Nature 566, 496–502. 30787437 10.1038/s41586-019-0969-x PMC6434952

[R8] Choi HMT, Beck VA, and Pierce NA (2014). Next-generation in situ hybridization chain reaction: higher gain, lower cost, greater durability. ACS Nano 8, 4284–4294. 24712299 10.1021/nn405717p PMC4046802

[R9] Cruciat CM, Ohkawara B, Acebron SP, Karaulanov E, Reinhard C, Ingelfinger D, Boutros M, and Niehrs C (2010). Requirement of prorenin receptor and vacuolar H+-ATPase-mediated acidification for Wnt signaling. Science 327, 459–463. 20093472 10.1126/science.1179802

[R10] Curran K, Lister JA, Kunkel GR, Prendergast A, Parichy DM, and Raible DW (2010). Interplay between Foxd3 and Mitf regulates cell fate plasticity in the zebrafish neural crest. Dev. Biol. 344, 107–118. 20460180 10.1016/j.ydbio.2010.04.023 PMC2909359

[R11] Dottori M, Gross MK, Labosky P, and Goulding M (2001). The winged-helix transcription factor Foxd3 suppresses interneuron differentiation and promotes neural crest cell fate. Development 128, 4127–4138. 11684651 10.1242/dev.128.21.4127

[R12] Dupin E, Calloni GW, Coelho-Aguiar JM, and Le Douarin NM (2018). The issue of the multipotency of the neural crest cells. Dev. Biol. 444, S47–S59. 29614271 10.1016/j.ydbio.2018.03.024

[R13] EauClaire SF, Cui S, Ma L, Matous J, Marlow FL, Gupta T, Burgess HA, Abrams EW, Kapp LD, Granato M, (2012). Mutations in vacuolar H+ -ATPase subunits lead to biliary developmental defects in zebrafish. Dev. Biol. 365, 434–444. 22465374 10.1016/j.ydbio.2012.03.009 PMC3337356

[R14] Force A, Lynch M, Pickett FB, Amores A, Yan YL, and Postlethwait J (1999). Preservation of duplicate genes by complementary, degenerative mutations. Genetics 151, 1531–1545. 10101175 10.1093/genetics/151.4.1531 PMC1460548

[R15] Germanguz I, Lev D, Waisman T, Kim C-H, and Gitelman I (2007). Four *twist* genes in zebrafish, four expression patterns. Dev. Dyn. 236, 2615–2626. 17685477 10.1002/dvdy.21267

[R16] Hopwood ND, Pluck A, and Gurdon JB (1989). A Xenopus mRNA related to Drosophila twist is expressed in response to induction in the mesoderm and the neural crest. Cell 59, 893–903. 2590945 10.1016/0092-8674(89)90612-0

[R17] Ichihara A, and Yatabe MS (2019). The (pro)renin receptor in health and disease. Nat. Rev. Nephrol. 15, 693–712. 31164719 10.1038/s41581-019-0160-5

[R18] Ishii M, Merrill AE, Chan YS, Gitelman I, Rice DPC, Sucov HM, and Maxson RE Jr. (2003). Msx2 and Twist cooperatively control the development of the neural crest-derived skeletogenic mesenchyme of the murine skull vault. Development 130, 6131–6142. 14597577 10.1242/dev.00793

[R19] Jacomy M, Venturini T, Heymann S, and Bastian M (2014). ForceAtlas2, a continuous graph layout algorithm for handy network visualization designed for the Gephi software. PLoS ONE 9, e98679. 24914678 10.1371/journal.pone.0098679 PMC4051631

[R20] Ji Y, Hao H, Reynolds K, McMahon M, and Zhou CJ (2019). Wnt signaling in neural crest ontogenesis and oncogenesis. Cells 8, 1173. 10.3390/cells8101173 PMC6829301 31569501

[R21] Kalcheim C, and Kumar D (2017). Cell fate decisions during neural crest ontogeny. Int. J. Dev. Biol. 61, 195–203. 28621417 10.1387/ijdb.160196ck

[R22] Kimmel CB, Ballard WW, Kimmel SR, Ullmann B, and Schilling TF (1995). Stages of embryonic development of the zebrafish. Dev. Dyn. 203, 253–310. 8589427 10.1002/aja.1002030302

[R23] Krispin S, Nitzan E, Kassem Y, and Kalcheim C (2010). Evidence for a dynamic spatiotemporal fate map and early fate restrictions of premigratory avian neural crest. Development 137, 585–595. 20110324 10.1242/dev.041509

[R24] La Manno G, Soldatov R, Zeisel A, Braun E, Hochgerner H, Petukhov V, Lidschreiber K, Kastriti ME, Lönnerberg P, Furlan A, (2018). RNA velocity of single cells. Nature 560, 494–498. 30089906 10.1038/s41586-018-0414-6 PMC6130801

[R25] Le Douarin N (1980). Migration and differentiation of neural crest cells. Curr. Top. Dev. Biol. 16, 31–85. 6110513 10.1016/s0070-2153(08)60153-2

[R26] Lignell A, Kerosuo L, Streichan SJ, Cai L, and Bronner ME (2017). Identification of a neural crest stem cell niche by spatial genomic analysis. Nat. Commun. 8, 1830. 29184067 10.1038/s41467-017-01561-w PMC5705662

[R27] Lister JA, Cooper C, Nguyen K, Modrell M, Grant K, and Raible DW (2006). Zebrafish Foxd3 is required for development of a subset of neural crest derivatives. Dev. Biol. 290, 92–104. 16364284 10.1016/j.ydbio.2005.11.014

[R28] Lukoseviciute M, Gavriouchkina D, Williams RM, Hochgreb-Hagele T, Senanayake U, Chong-Morrison V, Thongjuea S, Repapi E, Mead A, and Sauka-Spengler T (2018). From pioneer to repressor: bimodal foxd3 activity dynamically remodels neural crest regulatory landscape in vivo. Dev. Cell 47, 608–628.e6. 30513303 10.1016/j.devcel.2018.11.009 PMC6286384

[R29] Lun ATL, McCarthy DJ, and Marioni JC (2016). A step-by-step workflow for low-level analysis of single-cell RNA-seq data with Bioconductor. F1000Res. 5, 2122. 27909575 10.12688/f1000research.9501.1 PMC5112579

[R30] McDowell IC, Manandhar D, Vockley CM, Schmid AK, Reddy TE, and Engelhardt BE (2018). Clustering gene expression time series data using an infinite Gaussian process mixture model. PLoS Comput. Biol. 14, e1005896. 29337990 10.1371/journal.pcbi.1005896 PMC5786324

[R31] McKinney MC, Fukatsu K, Morrison J, McLennan R, Bronner ME, and Kulesa PM (2013). Evidence for dynamic rearrangements but lack of fate or position restrictions in premigratory avian trunk neural crest. Development 140, 820–830. 23318636 10.1242/dev.083725 PMC3557777

[R32] Montero-Balaguer M, Lang MR, Sachdev SW, Knappmeyer C, Stewart RA, De La Guardia A, Hatzopoulos AK, and Knapik EW (2006). The mother superior mutation ablates foxd3 activity in neural crest progenitor cells and depletes neural crest derivatives in zebrafish. Dev. Dyn. 235, 3199–3212. 17013879 10.1002/dvdy.20959

[R33] Moro E, Ozhan-Kizil G, Mongera A, Beis D, Wierzbicki C, Young RM, Bournele D, Domenichini A, Valdivia LE, Lum L, (2012). In vivo Wnt signaling tracing through a transgenic biosensor fish reveals novel activity domains. Dev. Biol. 366, 327–340. 22546689 10.1016/j.ydbio.2012.03.023

[R34] Morrison JA, McLennan R, Wolfe LA, Gogol MM, Meier S, McKinney MC, Teddy JM, Holmes L, Semerad CL, Box AC, (2017). Single-cell transcriptome analysis of avian neural crest migration reveals signatures of invasion and molecular transitions. eLife 6, e28415. 29199959 10.7554/eLife.28415 PMC5728719

[R35] Picelli S, Faridani OR, Björklund ÅK, Winberg G, Sagasser S, and Sandberg R (2014). Full-length RNA-seq from single cells using Smart-seq2. Nat. Protoc. 9, 171–181. 24385147 10.1038/nprot.2014.006

[R36] Prasad MS, Charney RM, and García-Castro MI (2019). Specification and formation of the neural crest: perspectives on lineage segregation. Genesis 57, e23276. 30576078 10.1002/dvg.23276 PMC6570420

[R37] Raible DW, and Eisen JS (1994). Restriction of neural crest cell fate in the trunk of the embryonic zebrafish. Development 120, 495–503. 8162850 10.1242/dev.120.3.495

[R38] Robinson MD, McCarthy DJ, and Smyth GK (2010). edgeR: a Bioconductor package for differential expression analysis of digital gene expression data. Bioinformatics. 26, 139–140. 19910308 10.1093/bioinformatics/btp616 PMC2796818

[R39] Rocha M, Singh N, Ahsan K, Beiriger A, and Prince VE (2020). Neural crest development: insights from the zebrafish. Dev. Dyn. 249, 88–111. 31591788 10.1002/dvdy.122 PMC7273345

[R40] Saelens W, Cannoodt R, Todorov H, and Saeys Y (2019). A comparison of single-cell trajectory inference methods. Nat. Biotechnol. 37, 547–554. 30936559 10.1038/s41587-019-0071-9

[R41] Schilling TF, and Kimmel CB (1994). Segment and cell type lineage restrictions during pharyngeal arch development in the zebrafish embryo. Development 120, 483–494. 8162849 10.1242/dev.120.3.483

[R42] Schilling TF, Le Pabic P, and Hoffman TL (2010). Using transgenic zebrafish (*Danio rerio*) to study development of the craniofacial skeleton. J. Appl. Ichthyology 26, 183–186.

[R43] Simões-Costa M, and Bronner ME (2015). Establishing neural crest identity: a gene regulatory recipe. Development 142, 242–257. 25564621 10.1242/dev.105445 PMC4302844

[R44] Soldatov R, Kaucka M, Kastriti ME, Petersen J, Chontorotzea T, Englmaier L, Akkuratova N, Yang Y, Häring M, Dyachuk V, (2019). Spatiotemporal structure of cell fate decisions in murine neural crest. Science 364, eaas9536. 31171666 10.1126/science.aas9536

[R45] Soo K, O’Rourke MP, Khoo PL, Steiner KA, Wong N, Behringer RR, and Tam PPL (2002). Twist function is required for the morphogenesis of the cephalic neural tube and the differentiation of the cranial neural crest cells in the mouse embryo. Dev. Biol. 247, 251–270. 12086465 10.1006/dbio.2002.0699

[R46] Stemple DL, and Anderson DJ (1992). Isolation of a stem cell for neurons and glia from the mammalian neural crest. Cell 71, 973–985. 1458542 10.1016/0092-8674(92)90393-q

[R47] Stewart RA, Arduini BL, Berghmans S, George RE, Kanki JP, Henion PD, and Look AT (2006). Zebrafish foxd3 is selectively required for neural crest specification, migration and survival. Dev. Biol. 292, 174–188. 16499899 10.1016/j.ydbio.2005.12.035

[R48] Stuart T, Butler A, Hoffman P, Hafemeister C, Papalexi E, Mauck WM 3rd, Hao Y, Stoeckius M, Smibert P, and Satija R (2019). Comprehensive integration of single-cell data. Cell 177, 1888–1902.e21. 31178118 10.1016/j.cell.2019.05.031 PMC6687398

[R49] Trapnell C, Cacchiarelli D, Grimsby J, Pokharel P, Li S, Morse M, Lennon NJ, Livak KJ, Mikkelsen TS, and Rinn JL (2014). The dynamics and regulators of cell fate decisions are revealed by pseudotemporal ordering of single cells. Nat. Biotechnol. 32, 381–386. 24658644 10.1038/nbt.2859 PMC4122333

[R50] Tuttle AM, Hoffman TL, and Schilling TF (2014). Rabconnectin-3a regulates vesicle endocytosis and canonical Wnt signaling in zebrafish neural crest migration. PLoS Biol. 12, e1001852. 24802872 10.1371/journal.pbio.1001852 PMC4011682

[R51] Van den Berge K, Roux de Bézieux H, Street K, Saelens W, Cannoodt R, Saeys Y, Dudoit S, and Clement L (2020). Trajectory-based differential expression analysis for single-cell sequencing data. Nat Commun. 11, 1201. 32139671 10.1038/s41467-020-14766-3 PMC7058077

[R52] Vaquerizas JM, Kummerfeld SK, Teichmann SA, and Luscombe NM (2009). A census of human transcription factors: function, expression and evolution. Nat. Rev. Genet. 10, 252–263. 19274049 10.1038/nrg2538

[R53] Vibert L, Aquino G, Gehring I, Subkankulova T, Schilling TF, Rocco A, and Kelsh RN (2017). An ongoing role for Wnt signaling in differentiating melanocytes in vivo. Pigment Cell Melanoma Res. 30, 219–232. 27977907 10.1111/pcmr.12568 PMC5360516

[R54] Wang S, Karikomi M, MacLean AL, and Nie Q (2019). Cell lineage and communication network inference via optimization for single-cell transcriptomics. Nucleic Acids Res. 47, e66. 30923815 10.1093/nar/gkz204 PMC6582411

[R55] Westerfield M (2000). The Zebrafish Book. A Guide for the Laboratory Use of Zebrafish (Danio rerio) (University of Oregon).

[R56] Weston JA (1970). The migration and differentiation of neural crest cells. In Advances in Morphogenesis, Abercrombie M, Brachet J, and King TJ, eds. (Elsevier), pp. 41–114. 10.1016/b978-0-12-028608-9.50006-5 4906187

[R57] Willems B, Tao S, Yu T, Huysseune A, Witten PE, and Winkler C (2015). The Wnt co-receptor Lrp5 is required for cranial neural crest cell migration in zebrafish. PLoS ONE 10, e0131768. 26121341 10.1371/journal.pone.0131768 PMC4486457

[R58] Williams RM, Candido-Ferreira I, Repapi E, Gavriouchkina D, Senanayake U, Ling ITC, Telenius J, Taylor S, Hughes J, and Sauka-Spengler T (2019). Reconstruction of the global neural crest gene regulatory network in vivo. Dev. Cell 51, 255–276.e7. 31639368 10.1016/j.devcel.2019.10.003 PMC6838682

[R59] Wolf FA, Hamey FK, Plass M, Solana J, Dahlin JS, Göttgens B, Rajewsky N, Simon L, and Theis FJ (2019). PAGA: graph abstraction reconciles clustering with trajectory inference through a topology preserving map of single cells. Genome Biol. 20, 59. 30890159 10.1186/s13059-019-1663-x PMC6425583

[R60] Wu RS, Lam II, Clay H, Duong DN, Deo RC, and Coughlin SR (2018). A rapid method for directed gene knockout for screening in G0 zebrafish. Dev. Cell 46, 112–125.e4. 29974860 10.1016/j.devcel.2018.06.003

[R61] Zhang AW, O’Flanagan C, Chavez EA, Lim JLP, Ceglia N, McPherson A, Wiens M, Walters P, Chan T, Hewitson B, (2019). Probabilistic cell-type assignment of single-cell RNA-seq for tumor microenvironment profiling. Nat. Methods 16, 1007–1015. 31501550 10.1038/s41592-019-0529-1 PMC7485597

