# Supplementary material for: Single-cell transcriptomic analysis of zebrafish cranial neural crest reveals spatiotemporal regulation of lineage decisions during development
Source: Cell Rep. Author manuscript; Available in PMC 2022 Jan 7. (PMC8741273; doi:10.1016/j.celrep.2021.110140)
Supplement: 1 [file NIHMS1766435-supplement-1.pdf]

Cell Reports, Volume *37*

Supplemental information

Single-cell transcriptomic analysis of zebrafish cranial neural crest reveals spatiotemporal regulation of lineage decisions during development

David Tatarakis, Zixuan Cang, Xiaojun Wu, Praveer P. Sharma, Matthew Karikomi, Adam L. MacLean, Qing Nie, and Thomas F. Schilling

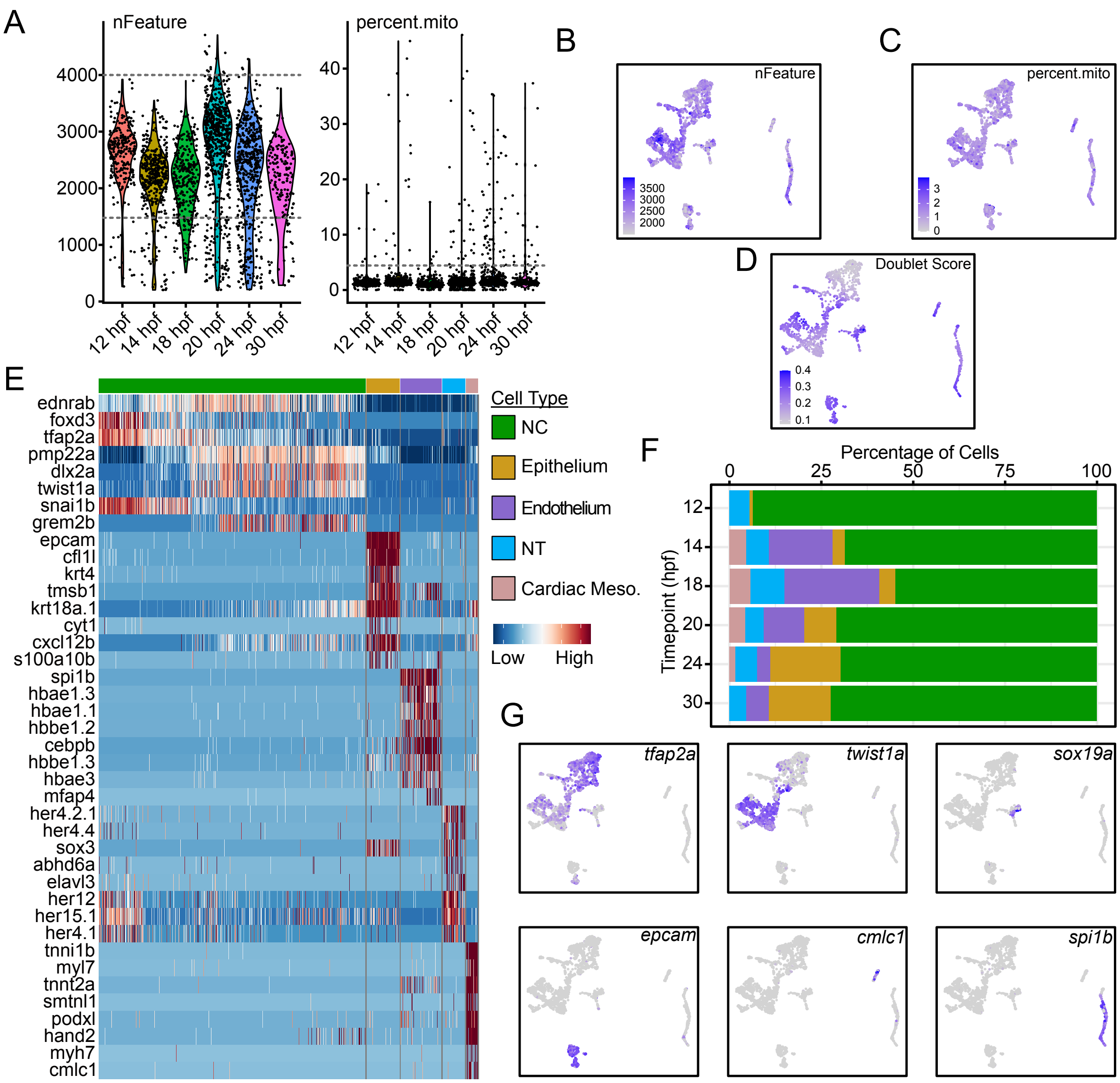

Figure S1: Expression profiles of non-NC cell types, Related to Figure 1. A) Violin plots showing QC metrics for whole arch single-cell object. Cells were excluded with >4000 features or <1500 features detected (dashed lines). Cells were excluded with >4% of reads mapped to mitochondrial genes (dashed line). **B)** Feature plot showing nFeature per cell in filtered whole arch object. **C)** Feature plot showing percent mitochondrial reads per cell in filtered whole arch object. **D)** Feature plot showing Doublet detection scores generating using DoubletFinder. **E)** Heatmap showing top marker genes in each cell type in Fig. 1C. **F)** Stacked barplots showing the number of each cell type recovered in each time point. **G)** Feature plots showing expression of strong marker genes for each cell type overlayed with UMAP.

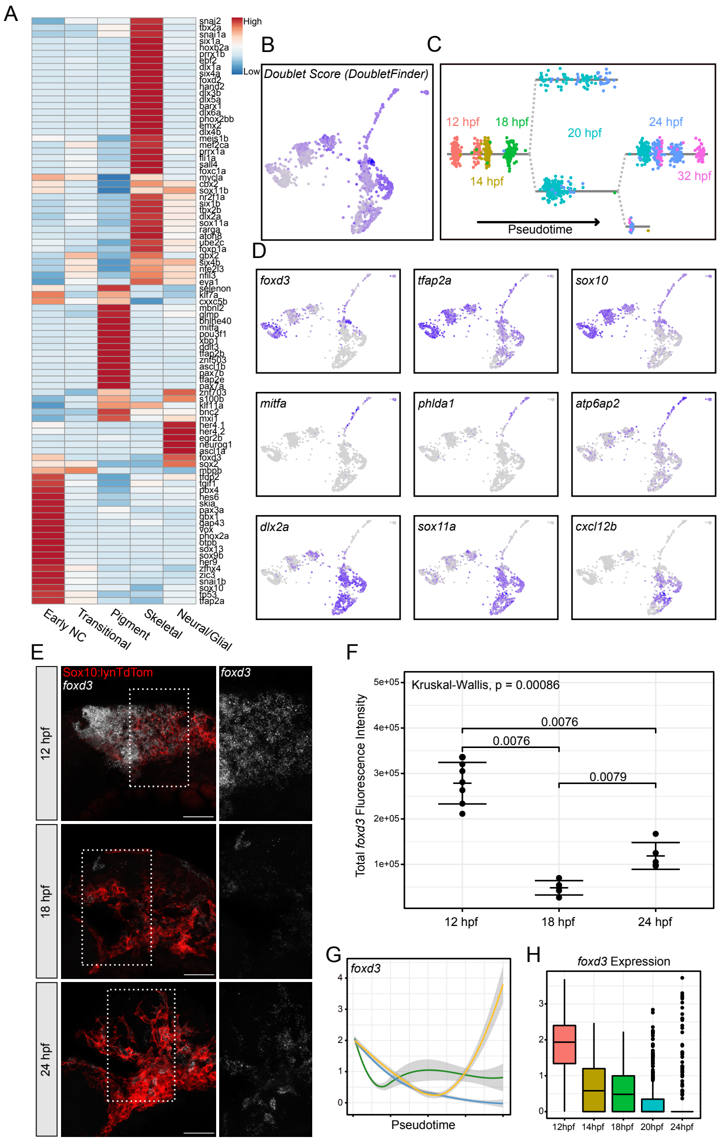

Figure S2: Marker gene profiles of NC lineages and in vivo temporal expression patterns of *foxd3*, Related to Figure 3. A) Heatmap showing average expression of top lineage markers in each NC subtype. **B)** Doublet detection scores generating using DoubletFinder overlayed with ForceAtlas (FA) Embedding. **C)** Dendroplot with cells ordered according to pseudotime and colored according to real developmental time point identity. **D)** Feature plots showing relative expression (dark purple indicates high levels) of lineage markers overlayed with FA embedding. **E)** Confocal micrographs of isHCR for *foxd3* expression (white) at 12, 18, and 24 hpf in *tg(sox10:lynTdTomato)* embryos where NC plasma membranes are marked by tdTomato (red). Enlarged region is the PA1 migratory stream. **F)** Quantification of *foxd3* expression from isHCR images in PA1 using corrected total fluorescence intensity (Y axis) at 12 hpf (n = 7 embryos, mean = 278640), 18 hpf (n = 5 embryos, mean = 48478), and 24 hpf (n = 5 embryos, mean = 118584). Dots represent means in individual embryos; lines represent means within conditions. Error bars represent mean ±SD. **G)** Local regression graph for *foxd3* expression levels (Y axis) in pigment (green), glial (yellow) and skeletal (blue) progenitors across pseudotime (X axis). Lines indicate moving averages for each branch. **H)** Barplots showing expression levels of *foxd3* (Y axis) across developmental time points (X axis). Lines indicate means, boxes indicate IQR, whiskers indicate IQR*1.5, points indicate outliers. For micrographs, Scale bars = 50µm.

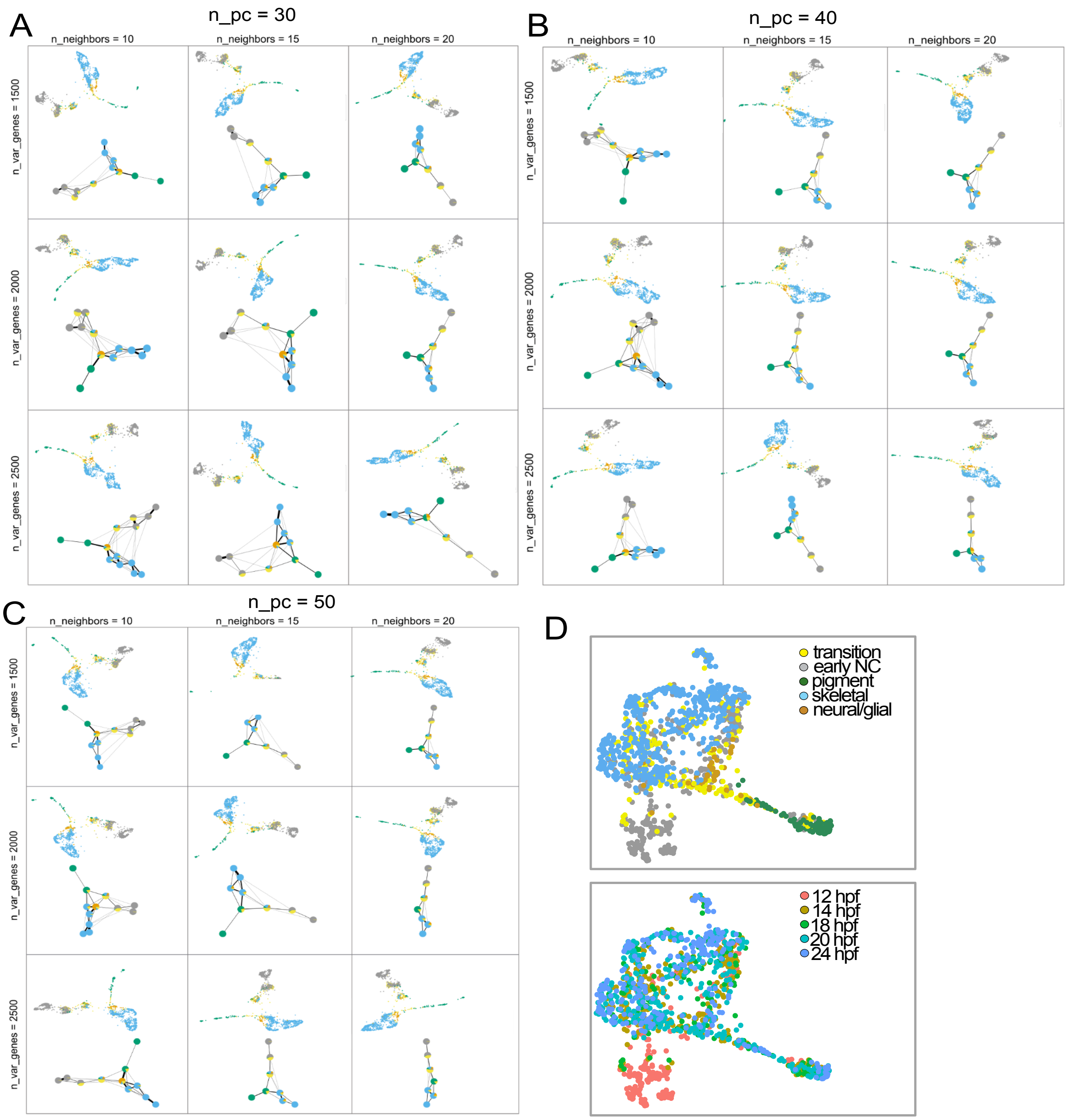

Figure S3: Temporal NC trajectories inferred from scRNA-seq are robust, Related to Figure 3. A-C) Temporal trajectories and the coarse graph of cell type compositions obtained using different parameter combinations. Number of principal components (n_pc), number of neighbors (n_neighbors), and number of variable genes (n_var_genes). Colors match lineage colors in Fig S3D. **D)** UMAPs showing integration by CCA of each library to remove timepoint-specific gene expression differences.

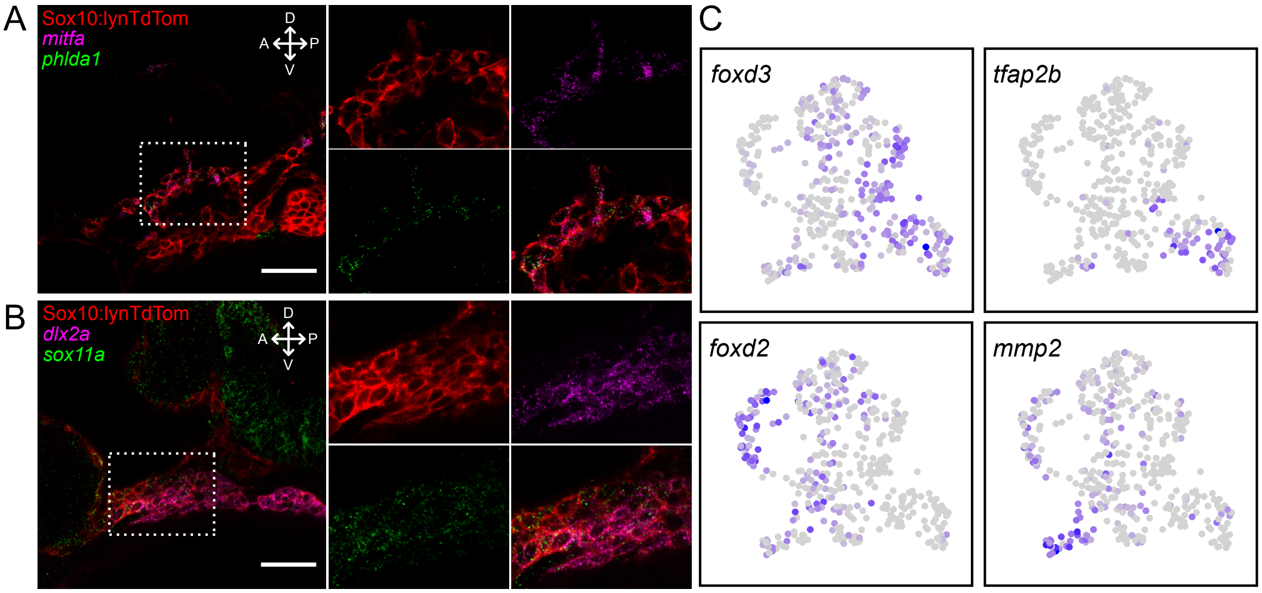

Figure S4: Additional marker gene analysis of skeletal and pigment lineages at 24 hpf in vivo and at 18 hpf in silico, Related to Figure 4. Confocal micrographs of isHCRs co-labeled for **A)** phlda1 (green) and **B)** mitfa (magenta) in putative pigment progenitors (upper panels) and sox11a (green) and dlx2a (magenta) in putative skeletal progenitors (lower panels) at 24 hpf in *tg(sox10:lynTdTomato)* embryos where NC plasma membranes are marked by tdTomato (red). **C)** Feature plots showing expression of 4 marker genes in 18 hpf neural crest.

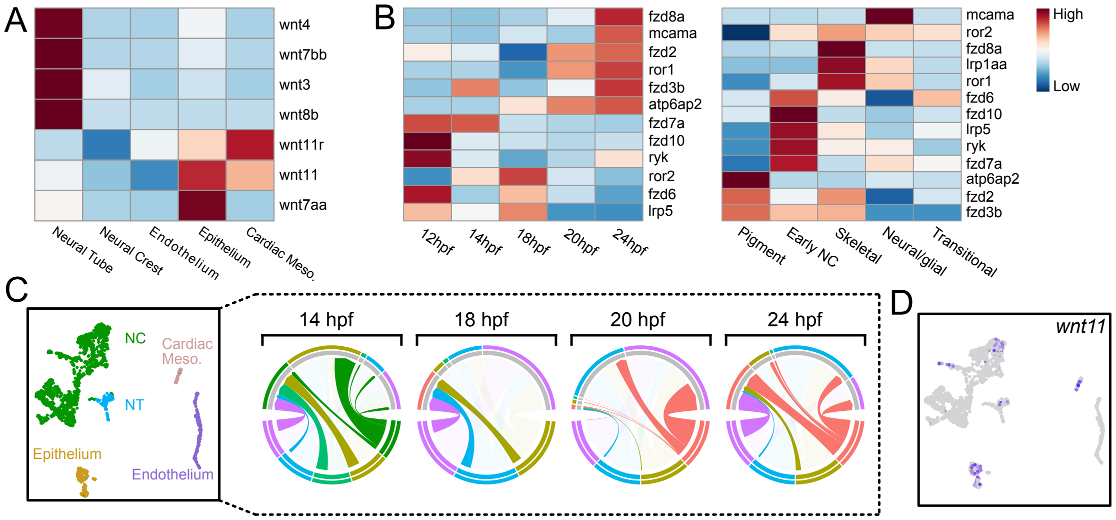

Figure S5: Cell-cell signaling analysis suggests changes in source of Wnt signaling across development in the NC, Related to Figure 6. A) Heatmap showing average expression of Wnt ligands in each cell type in the single cell timeline. **B)** Heatmaps showing average expression of Wnt receptors in NC cells both at different timepoints and in different lineages. **C)** Signaling analysis for cell-cell communication between NC cells and other cell types through SoptSC, color code corresponding to Fig. 1C. The bottom half of each circos plot represents signal-sending clusters; the top half represents signal-receiving clusters. Arrows are colored according to signal-sending cell type. Width of arrows indicates probability of a signaling event between the two cell types. **D)** Feature plot showing expression of *wnt11* in all cell types except endothelial, with NC expression confined mostly to early NC cells and pigment cells.

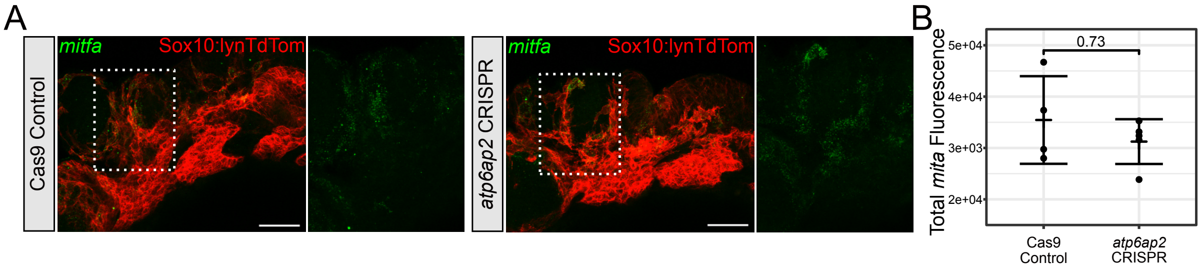

Figure S6: isHCR of mitfa reveals pigment progenitors are specified in atp6ap6 crispants, Related to Figure 6. A) Confocal micrographs of isHCR showing *mitfa* expression (green) at 24 hpf in a Cas9-injected control (left panel) and atp6ap2 CRISPR-injected embryo (right panel) in *tg(sox10:lynTdTomato)* transgenics where NC plasma membranes are marked by tdTomato (red). **B)** Quantification of *mitfa* expression using corrected total fluorescence intensity between control embryos (n = 4 embryos, mean = 35447) and *atp6ap2* CRISPR embryos (n = 5 embryos, mean = 31242). Wilcox p-value = 0.73. Dots represent means in individual embryos. Lines represent means within conditions. Error bars represent means ±SD. For micrographs, Scale bars = 50µm.
